# Fungal-fungal cocultivation alters secondary metabolites of marine fungi mediated by reactive oxygen species (ROS)

**DOI:** 10.1128/mbio.01447-25

**Published:** 2025-08-18

**Authors:** Yu Xiao, Yongqi Li, Yukun Cui, Paiyao Ji, Zhizhen Zhang, Jiasong Fang, Xi Yu

**Affiliations:** 1 Shanghai Engineering Research Center of Hadal Science and Technology, College of Oceanography and Ecological Science, Shanghai Ocean University 74595, Shanghai, China; 2 Ocean College, Zhoushan Campus, Zhejiang University 601090, Zhoushan, China; University of Melbourne, Melbourne, Victoria, Australia

**Keywords:** fungi, cocultivation, gene clusters, secondary metabolites, oxidative stress

## Abstract

Microorganisms form complex ecological networks of interactions, with secondary metabolites (SMs) playing a crucial role in microbial communication. This study explored the cocultivation of marine fungi *Alternaria alternata* and *Nigrospora sphaerica*, uncovering significant SM production changes. The biosynthetic gene clusters (BGCs) and transcription factor *aohR* in marine *A. alternata* were activated, leading to enhanced production of alternariol (AOH), a compound inhibiting fungal competitors, whereas the activation was not present in the coculture of terrestrial strains of *A. alternata*. Transcriptomic and metabolic analyses further revealed that reactive oxygen species (ROS) play a crucial role as signaling molecules in these marine fungal interactions. Elevated ROS levels triggered the upregulation of oxidative stress response genes in marine *A. alternata*, regulating the expression of *aohR*, which subsequently induced the biosynthesis of AOH. These findings highlight a unique defensive strategy in marine fungi, offering insights into their ecological roles and potential antibiotic development.

**IMPORTANCE:**

Marine fungi are an important source of natural products, and many marine-derived secondary metabolites have been found to exhibit significant bioactivities, including antibacterial, antiviral, and anticancer properties. Cocultivation is a simple and effective method for discovering bioactive compounds. However, whether marine-derived and terrestrial-derived microorganisms exhibit differential response mechanisms under co-culture conditions remains unexplored. Our study details how cocultivation of marine fungi triggers the activation of biosynthetic gene clusters (BGCs) and leads to an increase in active secondary metabolites (SMs), which may contribute to the future development of antibiotics or anticancer drugs. Furthermore, our findings reveal that reactive oxygen species (ROS) function as specific signaling molecules in marine fungal-fungal interactions, offering novel insights into the evolutionary strategies of fungi in marine ecosystems.

## INTRODUCTION

The ocean is the cradle of life, covering a vast area of the Earth’s surface and containing 80% of the planet’s biological resources. Fungi are ubiquitous in all marine habitats, occurring in a range of environments, from hydrothermal vents, deep-sea sediments, and Antarctic ice to surface intertidal waters, salt marshes, and beaches. They coexist with a diverse array of other marine organisms, including algae, corals, sponges, and other marine flora and fauna (1). They thrive in the marine biosphere and play an indispensable role in nutrient cycling within various ecosystems (2).

Microbial secondary metabolites are a diverse array of low-molecular-weight compounds produced by microorganisms to adapt to various environmental stresses (3, 4). The biosynthesis of each secondary metabolite is tightly regulated by complex control networks to cope with biotic and abiotic stresses in nature (5, 6). Fungi serve as important sources of secondary metabolites (SMs), which exhibit a vast array of chemical structures and have a multitude of potential applications, including drug discovery and the development of biopesticides (4, 7). However, in recent years, the number of newly discovered fungal SMs has significantly decreased. Fortunately, the increasing evidence suggests that microbial interactions are the driving force behind SM production (8, 9). Under simulated natural conditions, fungal cocultivation frequently results in enhanced production of known SMs or the reactivation of silent biosynthetic gene clusters (BGCs) to produce cryptic SMs for defensive purposes (7, 10, 11). Co-cultivation is a straightforward and effective approach to stimulate SM synthesis, aiming to discover bioactive metabolites or novel compounds. However, in most cases, the molecular mechanisms underlying the activation of BGCs during cocultivation remain largely elusive.

Different habitats have a significant impact on fungal survival, largely due to variations in intracellular reactive oxygen species (ROS) levels. Fungal adaptation to the environment is closely linked to the dynamic regulation of ROS to maintain redox homeostasis (12). Currently, research on ROS in fungi primarily focuses on plant pathogenic fungi. To survive oxidative bursts and successfully colonize their hosts, plant pathogenic fungi employ complex mechanisms to sense ROS, neutralize ROS, and prevent ROS-mediated damage (13, 14). Their strategies include the activation of antioxidant enzymes (such as superoxide dismutase and catalase), the thioredoxin system, glutathione biosynthesis, and DNA repair pathways (12, 15). In addition, ROS are not only toxic by-products of aerobic metabolism; they are also regarded as signaling molecules that regulate fungal physiological responses and developmental processes (16). They can induce the expression of defense-related genes, facilitate interspecific recognition, and trigger downstream responses such as SM production and programmed cell death (17–19).

Due to the extreme biotic and abiotic conditions in marine ecosystems, marine fungi have evolved various metabolic pathways to adapt to these harsh environments and produce specific bioactive compounds and chemical scaffolds (20). Secondary metabolites isolated from marine fungi have made significant contributions to the field of natural product research (21). However, studies on the relationship between secondary metabolites, cocultivation, and ROS of marine fungi remain relatively scarce. Although existing research indicates that it can affect secondary metabolite synthesis in marine fungi (22), the production of ROS by marine fungi under cocultivation conditions and its effects on secondary metabolism have not been adequately explored.

In order to deepen our understanding of fungal interactions in extreme environments, we conducted cocultivation experiments with marine fungi. The characterization of these interactions through SM profiling and bioactivity assays revealed that defense mechanisms triggered by interspecies interactions are pivotal factors influencing the alteration of SMs in marine *A. alternata*. This phenomenon was not found in terrestrial *Alternaria alternata*. Compound AOH was identified by high-performance liquid chromatography (HPLC) analysis and purification, which effectively inhibited the mycelial growth of *N. sphaerica* and exhibited potent antimicrobial and cytotoxic activities. Transcriptomic data and experimental data indicated that marine *A. alternata*, under changes in environmental pressure, activates the cellular defense system by accumulating ROS and transmitting damage signals intracellularly, thereby controlling the transcription factor regulation of SM production. This evidence suggests that unique signaling pathways in marine fungi play a critical role in stimulating the biosynthesis of secondary metabolites produced by *A. alternata*. Our study provides valuable insights into the regulatory mechanisms of marine fungal interactions and offers fresh perspectives for adjusting cocultivation methods to modulate secondary metabolism.

## RESULTS

### Coculture of A*. alternata* Z4 and *N. sphaerica* Z7 leads to the alteration of SMs

**Fig 1:**
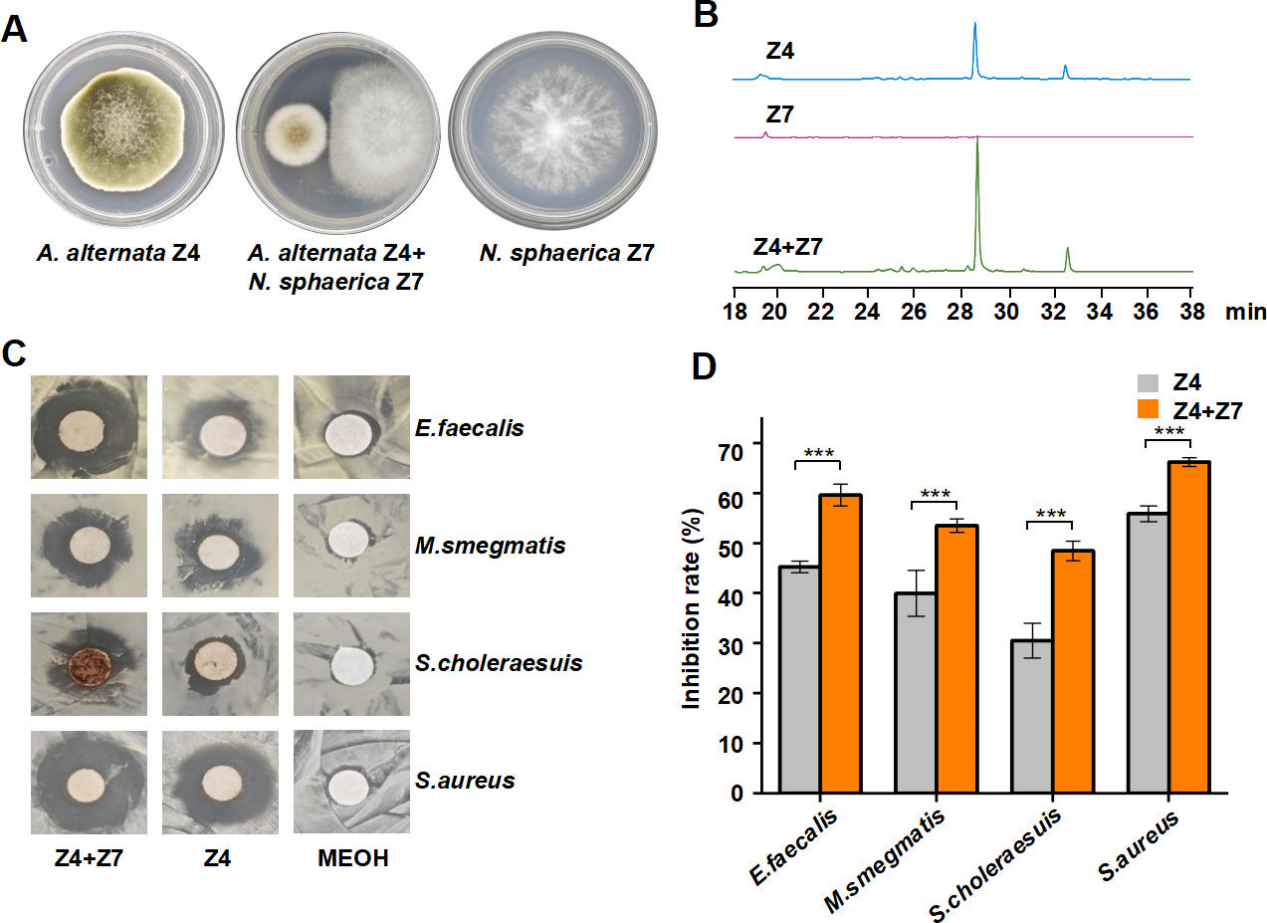
Cocultivation of *A. alternata* with *N. sphaerica* demonstrated alteration of secondary metabolites. (**A**) Growth of *A. alternata* Z4 and *N. sphaerica* Z7 strains on PDA plates under monocultivation and cocultivation conditions. (**B**) HPLC analysis of crude extracts from monocultivated and cocultivated strains. Ultraviolet (UV) absorptions at 220 nm are illustrated. Inhibition zones (**C**) and inhibition ratio (**D**) of crude extracts of *A. alternata* Z4 monoculture and coculture against four pathogenic bacteria. The results were expressed as mean ± standard deviation (SD). The experiment was repeated three times with similar results. The statistical significance of the coculture inhibitory activity compared to the control group was determined using the two-tailed Student's *t* test. *** represents *P* < 0.0001. Gray Z4, *orange* Z4 + Z7. Z4, *A. alternata* Z4; Z7, *N. sphaerica* Z7; Z4 + Z7, *A. alternata* Z4 + *N. sphaerica* Z7. Plotted with Origin 2022.

In our study, four fungal strains, *Alternaria alternata* Z4, *Nigrospora sphaerica* Z7, *Cyphellophora fusarioides,* and *Cladosporium colombiae*, were isolated from the abdominal contents of Antarctic fish from Zhongshan Station, Antarctica. These strains were identified through a combination of morphological characterization and ITS sequence analysis. In order to gain insights into the intricate relationships between marine fungi, we examined the interactions of four fungal strains on agar plates. Strain Z4 had no discernible impact on the growth of several representative marine isolates (Fig. S1A). However, it was able to inhibit the growth of *N. sphaerica* Z7, which formed a clear zone around its colonies on agar plates (Fig. 1A).

Since clear zones developed around the strain Z4 colonies, we hypothesized that strain Z4 inhibited the growth of *N. sphaerica* Z7, potentially via its secreted products. To provide further evidence, we extracted the SMs of strain monocultures and cocultures. In contrast to the monocultures of *A. alternata* Z4, a significant accumulation of compound 1 was detected during coculture with Z7 strains, as evidenced by HPLC analysis (Fig. 1B). This result suggested that the interaction between *A. alternata* Z4 and *N. sphaerica* Z7 led to an increase in the production of SMs production. The same phenomenon was not observed in the other fungal combinations (Fig. S1B). Indeed, the antifungal experiments also corroborated this finding. A clear zone of inhibition was detected upon addition of the cocultured crude extracts, whereas only a weak zone of inhibition was observed upon addition of the monocultured strain Z4. No zones of inhibition were produced in the region of the addition of the negative control methanol (MeOH) (Fig. S2), which is consistent with the trend observed in HPLC graphs. This suggests that cocultivation induced SM production in strain Z4, which effectively inhibited the growth of *N. sphaerica*. Given that compound 1 was the most abundant compound in the crude extract, it was hypothesized that strain Z4 secreted compound 1 to inhibit the growth of *N. sphaerica* Z7.

Moreover, the *in vitro* antibacterial assay showed that the crude extracts could inhibit the growth of a wide range of pathogenic bacteria (Fig. 1C). The crude extract of *A. alternata* cocultured with *N. sphaerica* Z7 showed antibacterial activity against *Enterococcus faecalis*, *Mycobacterium smegmatis*, *Salmonella choleraesuis,* and *Staphylococcus aureus* at 10 mg/mL, with an inhibition rate of 59.6%, 53.5%, 48.5% and 66.2%, respectively (Fig. 1D). The inhibition rates were higher than those observed in the monoculture, indicating that compound 1 may have good inhibitory activity against pathogenic bacteria and may have potential applications.

### Co-culture stimulated AOH production

**Fig 2:**
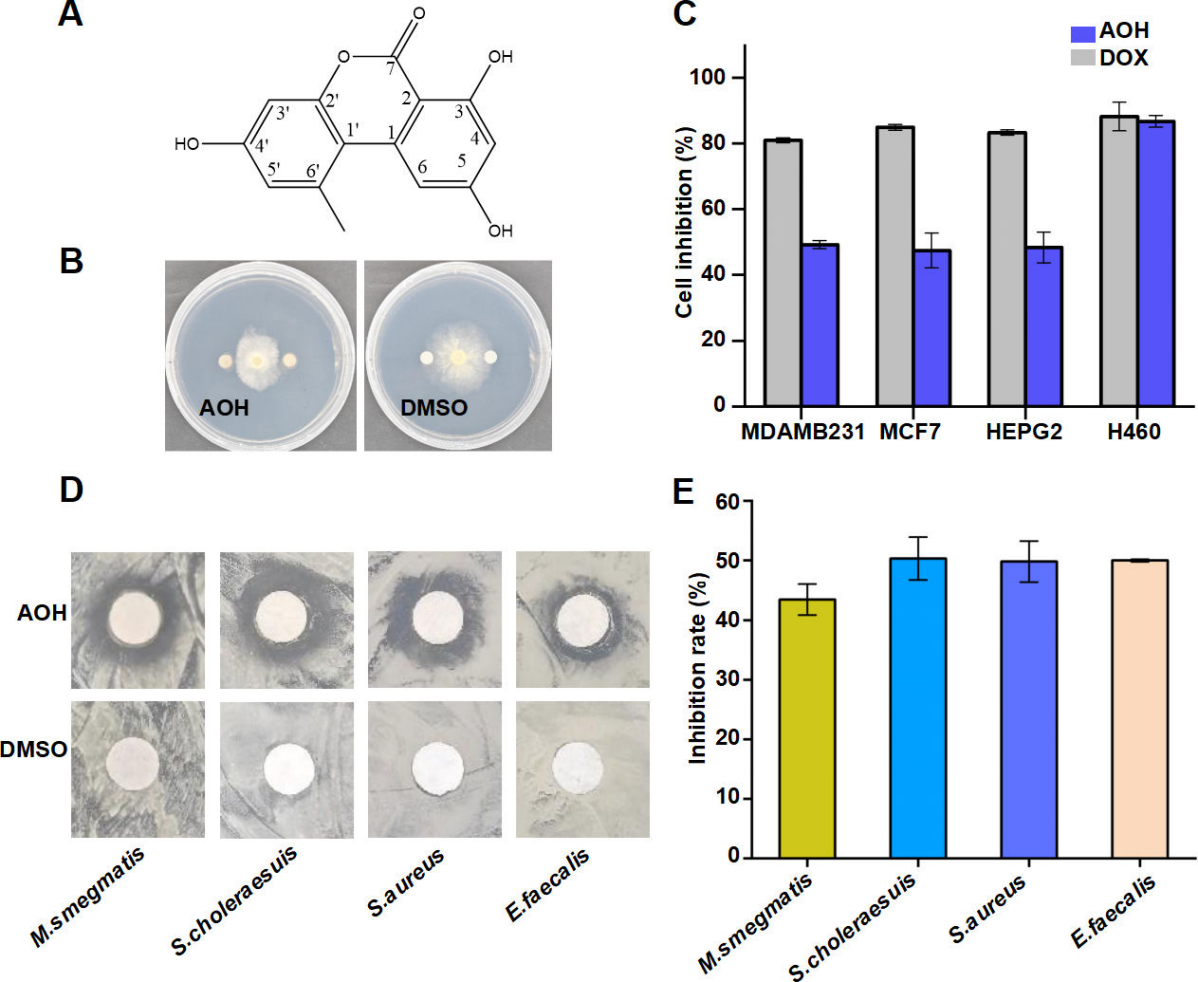
Biological activity of compound alternariol (AOH). (**A**) Chemical formula of compound AOH. ChemDraw 19.0. (**B**) Inhibition zone of *N. sphaerica* Z7 by AOH. (**C**) *In vitro* cytotoxicity activity of the AOH. *Blue* AOH; gray DOX. Inhibition zones (**D**) and inhibition ratio (**E**) of AOH against four pathogenic bacteria. The results were expressed as mean ± standard deviation (SD). Plotted with Origin 2022.

To further determine the upregulated metabolites, a scale-up coculture of *A. alternata* Z4 with *N. sphaerica* Z7 was performed in the rice medium for 30 days to obtain the crude extract. The crude extract was partitioned between MeOH and water. Column chromatography of the 70%–90% MeOH-soluble portion of the organic extract on the SHIMADZU LC-20AP HPLC system yielded compound 1 (8.2 mg) (Fig. 2A). Compound 1 was identified as alternariol (AOH) (23) based on 1H NMR, 13C NMR, and mass spectrometry data (Fig. S3 and S4; Table S2), which was corroborated by a comparison with previously published data. Its molecular formula was determined to be C_14_H_10_O_5_ (258.04). The filter paper disk diffusion method was employed to determine the inhibitory effect of alternariol on *N. sphaerica* Z7, with DMSO used as a negative control (Fig. 2B). The results showed the significant inhibition zones in the region of the filter paper disk where AOH was introduced, whereas the negative control exhibited no inhibitory effect. This finding is consistent with our hypothesis that coculture stimulates *A. alternata* Z4 to increase the production of AOH, which is subsequently employed to inhibit the growth of *N. sphaerica*.

AOH has been reported to inhibit cell proliferation and the growth of a variety of pathogenic bacteria (23, 24). The antitumor activity of alternariol was evaluated using a panel of human cancer cell lines, including NCI-H460, HepG2, MCF-7, and MDA-MB-231. AOH demonstrated a notable inhibitory effect on the growth of the four cell lines, with the inhibition values at 100 µM being 49.3% (MDA-MB-231), 48.5% (HepG2), 47.5% (MCF7), and 86.8% (NCI-H460), respectively (Fig. 2C). The positive control doxorubicin demonstrated efficacy in inhibiting these cell lines, with the inhibition values of 81.1% (MDA-MB-231), 83.4% (HepG2), 85.1% (MCF7), and 88.3% (NCI-H460) at 100 µM.

In addition, alternariol was tested *in vitro* for the antimicrobial activity against *S. choleraesuis*, *M. smegmatis*, *E. faecalis*, and *S. aureus*. The results demonstrated that alternariol exhibited antibacterial activity against all four pathogenic bacteria at 10 mg/mL (Fig. 2D), with inhibition rates of 50.3%, 43.4%, 50%, and 49.8%, respectively (Fig. 2E).

### Co-cultivation induces AOH synthesis and transcriptional activation of SM pathways in marine *A. alternata* Z4.

**Fig 3:**
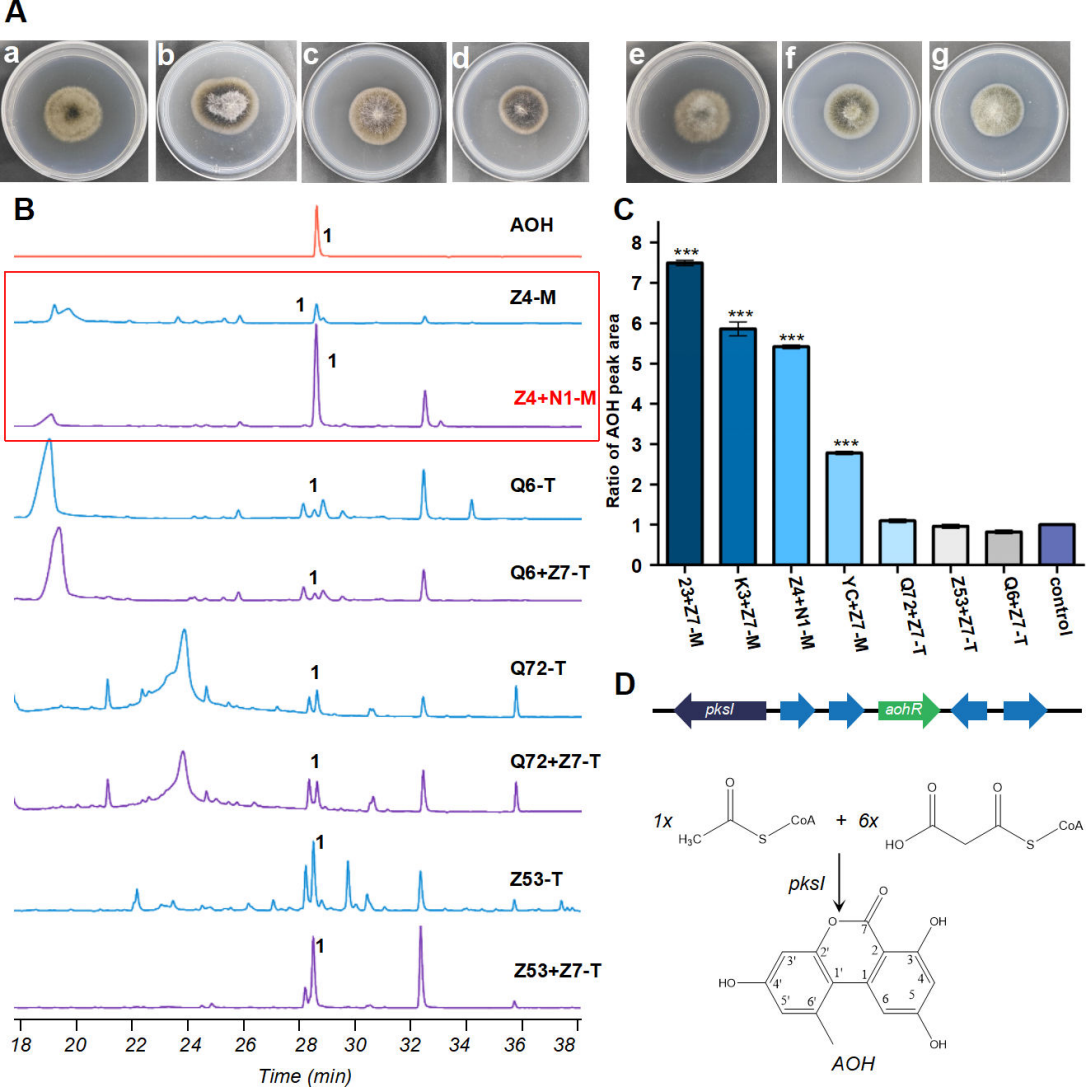
Identification and coculture secondary metabolite changes in *A. alternata* from different sources. (**A**) Plate morphology of *A. alternata* originating from marine and terrestrial areas, respectively, on PDA after 3 days of culture at 28°C. Marine: a, *A. alternata* Z4; b, *A. alternata* K3; c, *A. alternata* YC; d, *A. alternata* 23; terrestrial: e, *A. alternata* Q6; f, *A. alternata* Q72; g, *A. alternata* Z53. (**B**) HPLC profiles of crude extracts from monocultivated and cocultivated strains. Q6, *A. alternata* Q6; Z4 + N1, *A. alternata* Z4 + *N. sphaerica* N1; Q6 + Z7, *A. alternata* Q6 + *N. sphaerica* Z7. Q72, *A. alternata* Q72; Q72 + Z7, *A. alternata* Q72 + *N. sphaerica* Z7; Z53, *A. alternata* Z53; Z53 + Z7, *A. alternata* Z53 + *N. sphaerica* Z7. (**C**) Ratio of AOH peak area under *A. alternata* coculture from different sources compared to monoculture. Control, monoculture/monoculture; M, marine; T, terrestrial. The statistical significance of the ratio of AOH peak area compared to the control group was determined using the two-tailed Student's *t* test. * represents *P* < 0.05, ** represents *P* < 0.01, and *** represents *P* < 0.0001. Plotted with Origin 2022. (**D**) AOH biosynthetic gene cluster and biosynthetic pathway.

In a cocultivation experiment, we observed a significant phenomenon in which the marine fungus *A. alternata* interacts with *N. sphaerica*, resulting in a marked increase in the production of alternariol, which effectively inhibits the growth of *N. sphaerica*. This appears to be an effective survival strategy employed to resist the influence of competitors. To further understand this strategy, a comparative experiment was conducted using terrestrial *A. alternata* Q6, Q72, Z53, and *N. sphaerica* N1 as controls. The phylogenetic analysis and characterization of different sources of *A. alternata* are demonstrated in Fig. 3A and Fig. S5. HPLC analysis revealed that under cocultivation conditions, the alternariol production of terrestrial *A. alternata* Q6, Q72, and Z53 remained unchanged when cocultivated with *N. sphaerica* Z7, whereas marine *A. alternata* Z4 exhibited a significant 4.4-fold increase in alternariol production compared to monoculture when stimulated by the terrestrial *N. sphaerica* N1 (Fig. 3B and C).

The next question was whether the activation and response processes associated with SM production were specific in marine fungi. To analyze the generality of the stimulation of SM production in *A. alternata* from marine sources when cocultured with *N. sphaerica*, we further investigated the changes in SMs of three additional marine *A. alternata* strains isolated from different sediment depths (6,900 and 7,332 m) during coculture with *N. sphaerica*. The results showed that upon stimulation by *N. sphaerica*, the AOH production of marine *A. alternata* strains 23, K3, and YC in coculture increased by 6.4 ± 0.04 fold, 4.8 ± 0.17 fold, and 1.7 ± 0.03 fold (*n* = 3), respectively, compared to their monoculture counterparts. (Fig. S6; Fig. 3C). This result indicates that the presence of *N. sphaerica* can stably and effectively stimulate the synthesis of AOH in marine *A. alternata*.

To elucidate the molecular mechanisms underlying the coculture interaction, we selected monocultures of marine *A. alternata* Z4 and terrestrial *A. alternata* Q6, as well as cocultures of those with marine *N. sphaerica* Z7, for transcriptomic analysis.

Clean reads were obtained by removing adapters, sequences with > 10% N bases, and low-quality sequences (Phred score Q ≤ 5, > 50% of reads, Table S3). Principal component analysis of gene expression profiles in *A. alternata* revealed a distinct divergence between the monoculture and coculture groups (Fig. S7A and B). Compared to the monoculture, the RNA-seq analysis of coculture revealed 663 DEGs in the transcriptome of *A. alternata* Z4, including 422 upregulated genes and 241 downregulated genes (Figure S8A). The RNA-seq analysis of *A. alternata* Q6 culture revealed a total of 386 genes with significantly changed transcript levels, including 130 upregulated genes and 256 downregulated genes (Fig. S8B). Ten genes, including those that up or downregulated genes, were randomly selected and tested for expression profiling by qRT-PCR to validate the gene expression data obtained by RNA-seq. The results were consistent with the RNA-seq data (Fig. S9A and B), indicating the reliability and reproducibility of the sequencing results.

**Fig 4:**
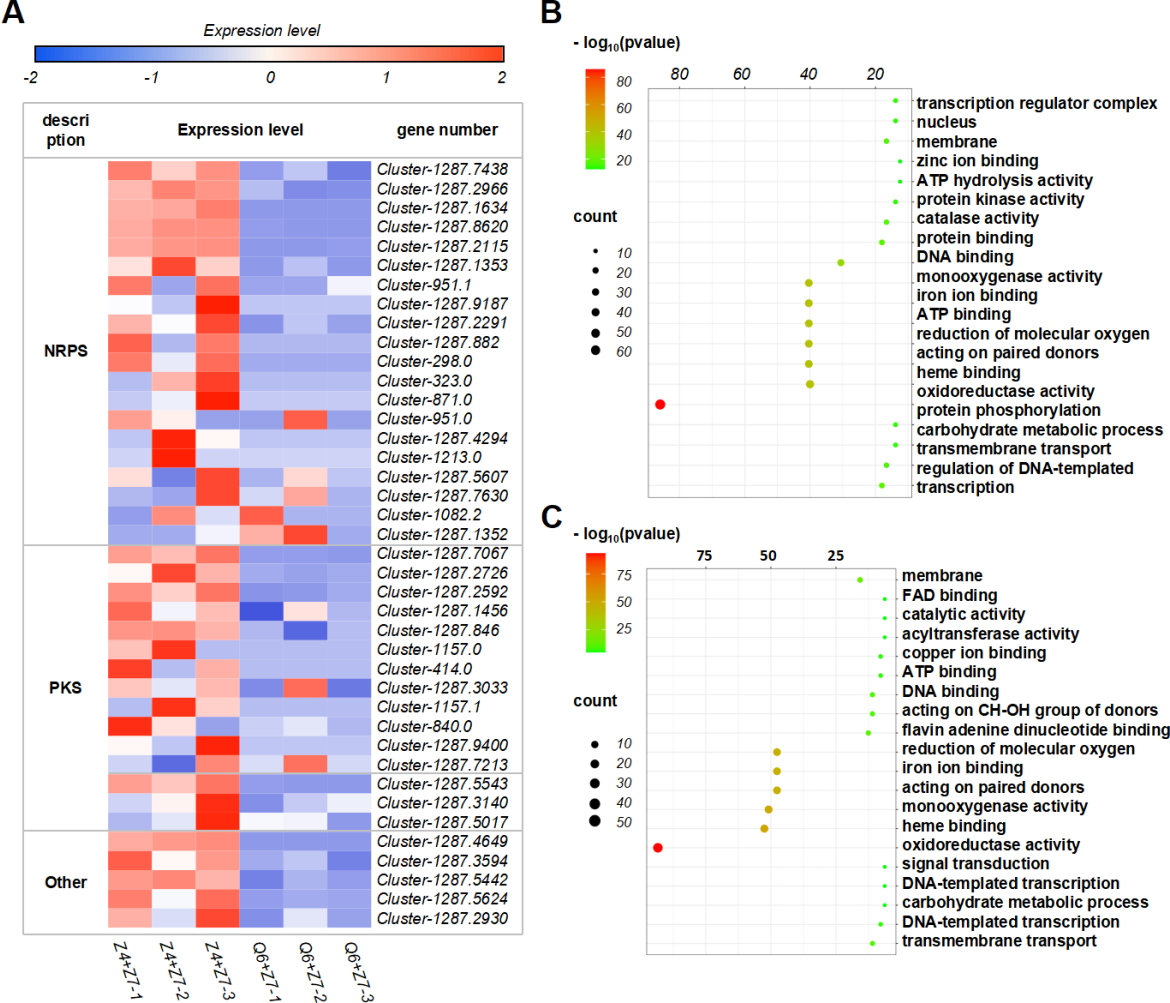
Widespread alterations of transcriptome and metabolism triggered by cocultivation of *A. alternata* and *N. sphaerica*. (**A**) Expression profiles of secondary metabolite biosynthetic genes during cocultivation. Data are for three biological replicates. Their corresponding types of BGCs referring to the published data are labeled on the left. NRPS, nonribosomal peptide synthetase; PKS, polyketide synthase; other, the biosynthetic pathway has not been elucidated yet. (**B**) Top 20 terms from GOs enriched from *A. alternata* Z4 cocultured DEGs. (**C**) Top 20 terms from GOs enriched from *A. alternata* Q6 cocultured DEGs.

We selected several annotation databases for in-depth analysis and identified a plethora of DEGs involved in secondary metabolism, which were responsible for the biosynthesis of chemical scaffolds, including polyketides, nonribosomal peptides, and terpenes. In accordance with the results of HPLC analysis, the polyketide synthase (PKS) gene *pksI*, located in the AOH biosynthetic gene cluster (Fig. 3D), showed upregulation in the cocultivated *A. alternata* Z4 (25), whereas its expression level was downregulated in terrestrial *A. alternata* Q6 (Table S4). LC-MS analysis revealed a strong correlation between alternariol production and the expression of BGCs during cocultivation. On the 3rd day of cocultivation, a signal indicative of the presence of compound AOH was detected in *A. alternata* Z4, while no signal was detected in *A. alternata* Q6 (Fig. S10A and B). Furthermore, an additional 16 SM biosynthetic genes were identified that exhibited a significant upregulation in marine *A. alternata* Z4, while genes were either downregulated or not expressed in terrestrial *A. alternata* Q6 (Table S4). Furthermore, a similar pattern of expression was observed in all three types of BGCs (nonribosomal peptide synthetase (NRPS), PKS, and other) that contained these backbone genes. (Fig. 4A). Overall, the transcriptomic and secondary metabolite data suggest that cocultivation with *N. sphaerica* triggers widespread changes in the expression levels of BGC genes in the marine *A. alternata* Z4, leading to modifications in the compound production. Among these changes, the most noteworthy is the increased production of the compound AOH, which points to a sophisticated pathway activation mechanism.

Transcriptional regulators play a crucial role in the complex network that controls the biosynthesis of secondary metabolites in fungi (26). The transcription factor *aohR* has been demonstrated to regulate AOH production in *A. alternata*, and *aohR* contains two specific domains. A GAL4-like Zn(II)2Cys6 (or C6 zinc) binuclear cluster DNA-binding domain and a fungal-specific transcription factor domain (25). It is noteworthy that the transcription factor *aohR* exhibited a notable degree of differential expression in strain Z4 during coculture, whereas it was not expressed in coculture with strain Q6 (Table S4), which aligns with the expression of the *pksI* gene cluster and the production of AOH. Taken together, our results suggest that coculture of *N. sphaerica* Z7 with marine *A. alternata* Z4 triggers the activation of SM pathways mediated by the transcription factor *aohR*.

### Overview of transcriptomes of *A. alternata* during interspecific interaction with *N. sphaerica* Z7

By screening the expression patterns of secondary metabolism gene clusters after coculture with different sources of *A. alternata*, we found extensive changes in gene expression associated with SM production that only occurred in marine fungi. This suggests that the surrounding environment exerts a considerable influence on the response strategy of these strains. To further substantiate the unique effect of cocultivation on marine *A. alternata*, we performed GO and KEGG enrichment analyses on DEGs of *A. alternata* Z4 and Q6 separately.

According to GO terms, the DEG functions were classified on the basis of a *P*-value ≤ 0.05. A comparison between cocultivation and monocultivation of *A. alternata* Z4 covered 20 principal functional categories, including four biological processes, 13 molecular functions, and three cellular components. The majority of DEGs, including those related to oxidoreductase activity and ion binding, were upregulated under cocultivation conditions (Fig. 4B). In contrast, a comparison between cocultivation and monocultivation of *A. alternata* Q6 showed that most DEGs exhibited downregulation, including five biological processes, 14 molecular functions, and one cellular component (Fig. 4C).

Using the KEGG database, enrichment analysis was performed on the top 20 pathways of DEGs, with a *P*-value ≤ 0.05. Compared to the conditions of Z4 monoculture, the genes involved in energy metabolism, including the TCA cycle, glycolysis, and oxidative phosphorylation, showed a general upregulation in the coculture of Z4 and Z7. In addition, genes associated with stress response pathways, including MAPK signaling, peroxisome, and DNA damage repair, showed heightened expression levels in *A. alternata* Z4 after cocultivation with *N. sphaerica* Z7 (Fig. S11A; Table S4). In contrast, KEGG pathways that were significantly enriched in strain Q6 after cocultivation treatment predominantly involved various amino acid metabolism, glycolysis, and oxidative phosphorylation, with the majority exhibiting downregulation (Fig. S11B; Table S4). These genes represent crucial pathways for maintaining cellular energy supply. A reduction in the expression of these genes could result in an inadequate energy supply, which in turn could impair the normal physiological activities and cell growth (27–30).

### Oxidative stress response under coculture conditions

In this study, we observed differential expression responses to oxidative stress in two strains of *A. alternata* originating from different sources. According to the KEGG database, genes related to ROS scavenging, such as *sod*, *katE*, *nox*, *GSR,* and *G6PD*, were upregulated to varying degrees during coculture with marine *A. alternata*. In addition, we observed the upregulation of several DNA repair-related genes, with *pol5*, *imp1*, *pold3*, *pole2*, and *pola2* showing more than a twofold increase. Similarly, the regulatory factors *hog1*, *skn7*, *ATM,* and *ATR* (Table S4; Fig. 6), which are associated with ROS and DNA repair signaling, also demonstrated an upward trend (31–33).

Surprisingly, these genes showed much lower activity during the interaction between terrestrial *A. alternata* and *N. sphaerica*. Genes involved in oxidative stress response and DNA repair showed varying degrees of downregulation or were not expressed at all (Table S4). It appeared that no defense signals were triggered in the presence of the invader. To validate this hypothesis, we determined the levels of ROS and mitochondrial membrane potential in both *A. alternata* strains under mono and cocultivation conditions.

**Fig 5:**
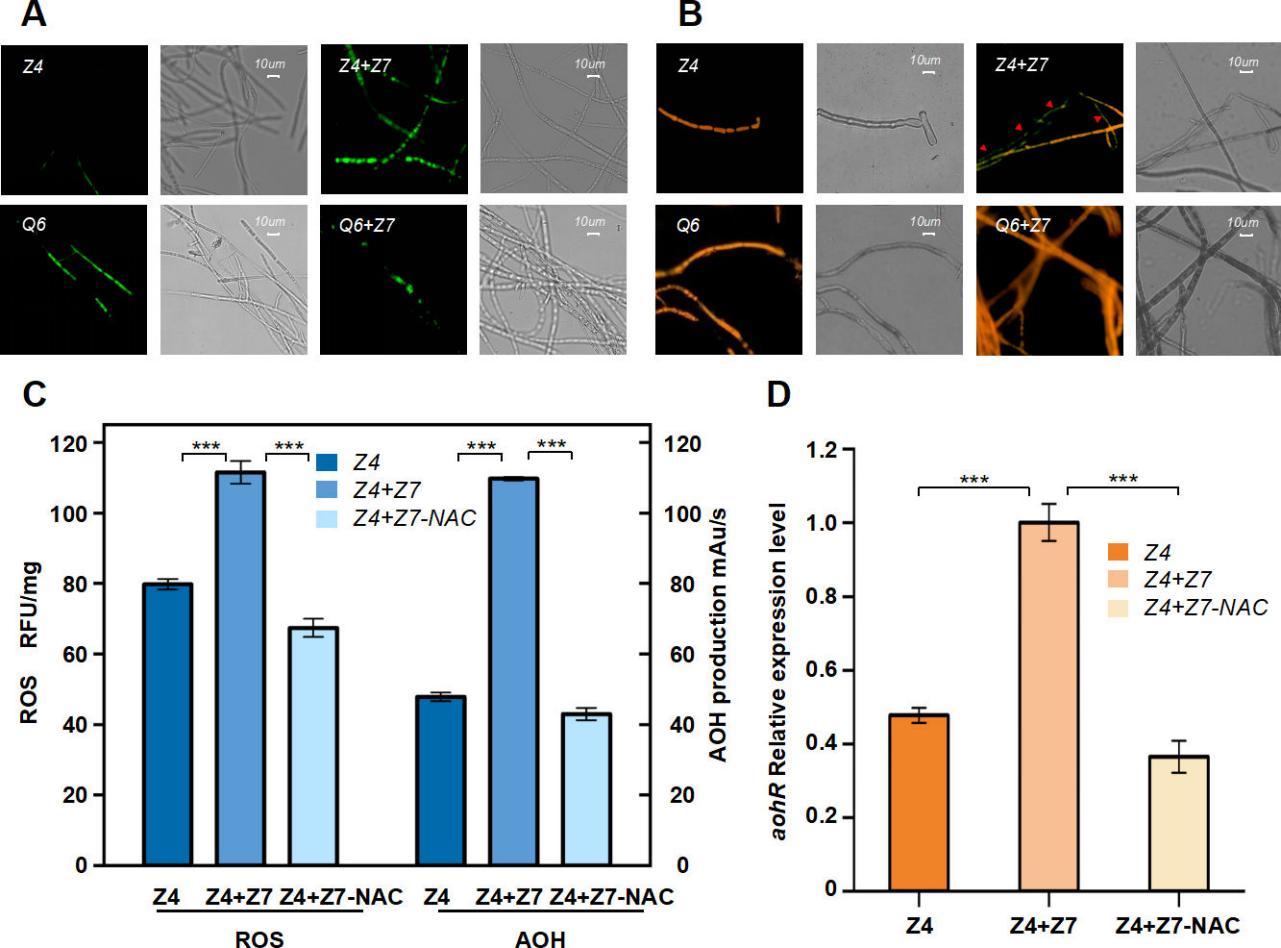
ROS was involved in the synthesis of alternariol (AOH) during *A. alternata* Z4-*N. sphaerica* Z7 interspecific interaction in separated coculture. (**A**) Hyphae of *A. alternata* Z4 stained with DCFH-DA under coculture conditions. (**B**) Hyphae of *A. alternata* Z4 stained with JC-1 under coculture conditions. Scale bar represents 10 µm. (**C**) ROS level and AOH production level of *A. alternata* Z4 in different culture conditions. (**D**) Relative expression level of *aohR* of *A. alternata* Z4 in different culture conditions. The results were expressed as mean ± standard deviation (SD). The experiments were repeated three times with similar results. The statistical significance of the fluorescence intensity of ROS, the peak area of alternariol, and *aohR* gene expression level compared with the control group were determined using a two-tailed Student's *t* test; *** represents *P* < 0.0001. Z4, *A. alternata* Z4; Z4 + Z7, *A. alternata* Z4 + *N. sphaerica* Z7; Z4 + Z7 NAC, *A. alternata* Z4 + *N. sphaerica* Z7 + NAC. Plotted with Origin 2022.

Our results indicate that under monoculture conditions, both strains exhibited a minimal accumulation of ROS, and there was no significant change in mitochondrial membrane potential. However, under coculture conditions, the marine strain *A. alternata* showed a significant accumulation of ROS, accompanied by a reduction in mitochondrial membrane potential. The fluorescent probe JC-1 exhibited a change in fluorescence from red to green (Fig. 5A and B). In contrast, only less ROS signal was detected in the cocultured terrestrial *A. alternata*, and there was no notable reduction in mitochondrial membrane potential. Based on these results, we speculate that when the external environment changes, marine *A. alternata* Z4 can rapidly receive this signal and activate oxidative stress to transmit the signal, thus enabling a more rapid adjustment of survival strategy. On the other hand, the terrestrial *A. alternata* Q6 shows a more conservative response to oxidative stress.

### ROS is involved in the synthesis of AOH in *A. alternata* Z4

Based on transcriptomic and LC-MS data, we hypothesize that oxidative stress induced by cocultivation with *N. sphaerica* triggers AOH production in *A. alternata* Z4, serving as a key signaling mechanism. To validate this hypothesis, we analyzed both the intracellular ROS levels and AOH production in *A. alternata* Z4 and Q6 during the cocultivation process. After 3 days of fungal culture, intracellular ROS levels were measured using a commercial assay kit. As shown in the data, the ROS concentration in *A. alternata* Z4 mono and cocultured cells was 79.8 rfu/mg and 111.53 rfu/mg, respectively, while the peak area for AOH in HPLC analysis was recorded at 48 mau/s and 109 mau/s (Fig. 5C). In contrast, the ROS concentrations in mono and cocultured Q6 cells were 67.3 rfu/mg and 59.4 rfu/mg, respectively, with AOH peak areas of 20 mau/s and 0 mau/s (Fig. S13A).

To further explore the role of oxidative stress in AOH production, we introduced N-acetylcysteine (NAC) and H_2_O_2_. NAC, an antioxidant and free radical scavenger, is known to enhance intracellular levels of glutathione (GSH). GSH plays a critical role in protecting cells from oxidative stress damage (34). NAC was added to the coculture system at the start, with a final concentration of 1 mM, and *N. sphaerica* was physically separated using a dialysis bag. After 3 days of coculture in the presence of NAC, ROS levels in *A. alternata* Z4 cells significantly dropped to 67 rfu/mg, while the AOH peak area simultaneously decreased to 41 mau/s (Fig. 5C). Similarly, in Q6 cells, both ROS levels and AOH peak areas decreased synchronously (Figure S12A). Furthermore, the expression level of *aohR* of the coculture system further supports our hypothesis. After the addition of NAC, the expression of the *aohR* gene was significantly downregulated 1.4-fold. (Fig. 5D). NAC effectively prevented the increase in intracellular ROS concentration and, as demonstrated by the results of the *aohR* transcription assay, which was involved in the regulation of the synthesis of AOH in *A. alternata* Z4 under coculture conditions. Our results demonstrated that NAC effectively prevented the increase in intracellular ROS and regulation of SMs, further supporting our hypothesis that ROS is a key signaling molecule for AOH induction.

After inducing oxidative stress with 1 mM H_2_O_2_, we observed a significant increase in intracellular ROS levels in both *A. alternata* strains Z4 and Q6. Along with the rise in ROS, the AOH production of strain Z4 increased by 2.6-fold, while that of strain Q6 increased by 1.2-fold (Fig. S12A ans S13A), indicating that ROS can significantly promote the synthesis of AOH and other secondary metabolites. Further transcriptome analysis showed that the expression level of the *aohR* gene was markedly upregulated after hydrogen peroxide treatment, with a 1.2-fold increase in strain Z4 and a 0.9-fold increase in strain Q6 (Fig. S12B and S13B). These results collectively support our hypothesis that ROS, acting as a signaling molecule, plays an important role in regulating secondary metabolite production in *A. alternata*.

## DISCUSSION

Researchers have been engaged in efforts to identify methods to activate silent BGCs. However, due to the inherent complexity of natural product biosynthesis, only a limited number of strategies have been developed to date, including treatments with epigenetic modifiers, mutagenesis, and genetic engineering (5). In addition to these methods, microbial cocultivation has been demonstrated to be an effective approach for driving the production of bioactive secondary metabolites (9). This approach simultaneously impacts multiple pathways, and the mechanisms of interaction between microorganisms may vary in different natural habitats, leading to varying effects on secondary metabolite production. In our study, when marine *A. alternata* and *N. sphaerica* were cocultured, *A. alternata* effectively inhibited the growth of *N. sphaerica* by increasing the production of compound AOH. However, this phenomenon was not observed in terrestrial strains of *A. alternata*. Notably, alternariol (AOH) is a common secondary metabolite produced by *Alternaria* species, and its biosynthesis is influenced by various environmental factors. Previous studies have shown that light conditions (35, 36), temperature fluctuations (37), pH changes, and the availability of carbon/nitrogen sources can influence its production (38, 39). Our study has shown that marine ecological interactions may also serve as a key regulator of AOH biosynthesis, thereby broadening our understanding of the regulatory mechanisms underlying secondary metabolism in *Alternaria*. Compared to terrestrial counterparts, marine fungi face greater challenges. Limited growth substrates, high salinity, and, in some cases, considerable hydrostatic pressure require marine microorganisms to adopt survival strategies that differ from those employed in typical environments, which enables them to gain a competitive advantage (1). Therefore, this phenomenon may be a unique feature of marine *A. alternata*. Transcriptomic analysis revealed that compound AOH and the *pksI* gene cluster exhibited concurrent changes. Furthermore, compared to monocultures, marine *A. alternata* showed a significant upregulation of 16 BGCs, while terrestrial-derived *A. alternata* demonstrated either downregulation or no expression. However, we did not detect corresponding increases in compound production other than AOH, which may be due to limitations in our current extraction methods and detector sensitivity. This represents a challenge that we aim to address in future experiments. Overall, the cocultivation of fungi in marine environments effectively enhances the biosynthesis of natural products and activates the BGCs responsible for AOH and other unidentified compounds. This specific response differs from that observed in terrestrial fungi. We believe that this effect could be exploited in the future to discover more biologically active natural products from marine environments.

The growth strategies of microorganisms can be significantly influenced by different environmental conditions, with the specific impact depending on factors such as microbial species, their genetic background, and the characteristics of the ecosystem (40). Our transcriptomic analysis revealed that the marine *A. alternata* exhibited upregulation in primary metabolic pathways such as glycolysis/gluconeogenesis, the TCA cycle, and oxidative phosphorylation. Additionally, pathways involved in the generation and breakdown of several amino acids were also upregulated. The direct competition for nutrients between the two fungi is responsible for the upregulation of glycolysis, TCA cycle, and oxidative phosphorylation pathways, which together contribute to the cellular energy supply and biosynthesis essential for maintaining normal cell function and growth (41). Compared to strain Z4, the terrestrial strain demonstrated a notable reduction in biomass and a general downregulation of genes related to energy metabolism under cocultivation conditions. This indicates that the downregulation of genes involved in amino acid metabolism, glycolysis, and oxidative phosphorylation may have a negative impact on fungal growth and metabolism, leading to a slowing of the growth rate, impaired cellular functions, and decreased cell viability. Our results indicated that marine fungi may rapidly occupy resources and inhibit the growth of competitors by increasing the activity of energy metabolic pathways and accelerating the biosynthesis of SMs. Moreover, the upregulation of energy metabolism pathways helps fungi maintain cellular homeostasis to cope with competitive pressure and environmental changes.

**Fig 6:**
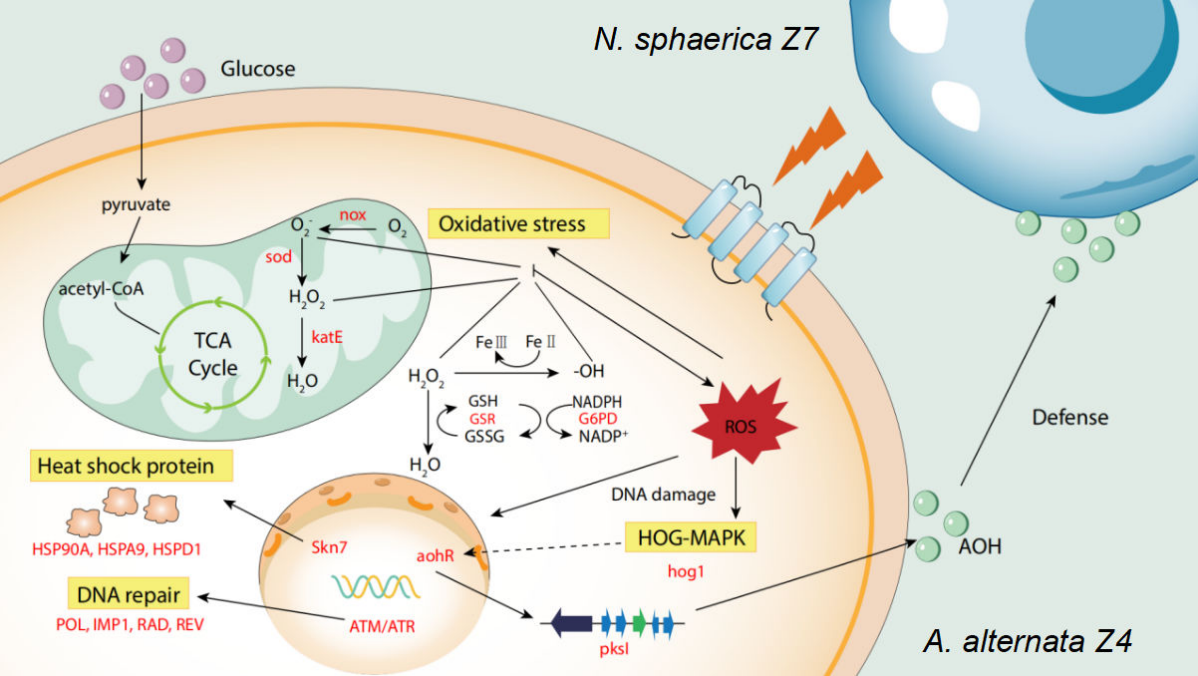
A conceptual model explains the mechanisms of coculture adaptation of *A. alternata* Z4 strain. ROS, reactive oxygen species; nox, NADPH (nicotinamide adenine dinucleotide phosphate) oxidase; sod, superoxide dismutase; katE, gene encoding catalase; GSR, glutathione reductase (NADPH); G6PD, glucose-6-phosphate 1-dehydrogenase; ATM, serine-protein kinase ATM; ATR, serine/threonine- protein kinase ATR. Skn7, osmolarity two-component system, response regulator SKN7. aohR, DNA-binding transcription factor activity; pksI, polyketide synthase; HSP90A, heat shock protein 90; HSPA9, heat shock protein 9; HSPD1, heat shock protein 60; POL, DNA polymerase; IMP1, mitochondrial inner membrane protease subunit 1; RAD, DNA repair protein RAD; REV, DNA repair protein REV. Red genes indicate genes upregulated under coculture conditions; hog1, p38 MAP kinase.

Antioxidant genes are ubiquitous in almost all microorganisms and are regarded as the core response to combat various environmental stresses (16). It has been reported that ROS play a central role in signal transduction during fungal-fungal interactions, leading to interspecies recognition and ultimately triggering fungal defense responses (17). In our study, we observed that marine strains of *A. alternata* exhibited superior growth characteristics, more sensitive responses to attack signals, and stronger antioxidant capabilities compared to their terrestrial counterparts during fungal interactions. This suggests that enhanced antioxidant capacity is a key factor in gaining a competitive advantage during these interactions. The correlation between ROS levels and AOH production indicates that oxidative stress may serve as a trigger for SM biosynthesis, highlighting the critical role of cellular stress responses in microbial metabolic regulation. Our findings also reveal a complex regulatory mechanism within marine *A. alternata*, where oxidative stress not only functions as a defense response but also acts as a stimulus for enhanced AOH biosynthesis during interspecies interactions. Previous studies have demonstrated a strong link between ROS and the *hog1* gene, a core MAPK of the high-osmolarity glycerol (HOG) pathway that mediates responses to oxidative stress in microorganisms. Extending these findings, our study reveals that ROS not only activate classical stress response pathways but also stimulate secondary metabolism. Specifically, ROS may act as early attack signals, coordinating the activation of the HOG-MAPK cascade and the transcription factor *aohR*, thereby inducing the *pksI* cluster for AOH production and inhibiting the growth of *N. sphaerica*. However, it should be noted that our conclusions are based on a limited number of strains, which may not fully capture the natural diversity of *A. alternata*. Future studies with expanded sampling across different oceanic zones, and more replicates are needed to validate these trends. It is worth further exploring why marine *A. alternata* exhibits a stronger and more specific response to *N. sphaerica*-induced oxidative signals. One possible explanation is that the long-term exposure to extreme conditions in the marine environment, such as high salinity and high pressure, has imposed persistent oxidative stress as a selective pressure and shaped unique ecological adaptations. This may include a heightened sensitivity to interspecies interactions, allowing marine strains to more effectively recognize and respond to biotic stress signals, such as those triggered by fungal co-cultivation. These adaptive strategies not only enhance the survival of marine fungi but may also confer a stronger ecological advantage, particularly in microbial communities where rapid detection and response to stimuli or stress signals from neighboring species are crucial. In summary, we propose a schematic model to illustrate the metabolic adaptation mechanisms of marine *A. alternata* in response to *N. sphaerica* stimulation (Fig. 6). The robust growth of *A. alternata* in marine environments is partially attributed to its strict regulation of ionic balance and enhanced antioxidant stress responses. Another critical aspect is the activation of BGCs that produce bioactive secondary metabolites, which serve as offensive weapons during direct microbial interactions, helping the marine strain secure an ecological niche in a harsh environment. This dual strategy—enhanced stress resilience and active production of defensive compounds—provides insights into how marine fungi adapt and thrive in their challenging habitats.

Our work confirms that marine fungi exhibit greater adaptability and survival capabilities, with ROS signaling being crucial for chemical signal transduction and survival defense during cocultivation. Co-cultivation with *N. sphaerica* stimulated ROS accumulation in marine *A. alternata* cells, which subsequently induced the expression of secondary metabolite gene clusters, ultimately increasing the production of the compound alternariol (AOH). Additionally, a significant upregulation of primary metabolism and oxidative stress response genes was observed. Our study reveals the unique defense mechanisms and survival strategies of marine *A. alternata* during its interaction with *N. sphaerica*. This cross-species interaction not only highlights the metabolic linkage between microorganisms but also contributes to the understanding of the ecological mechanisms of fungi in marine environments. Understanding this interaction mechanism will help further explore and utilize microbial cocultivation systems to optimize the production of secondary metabolites from marine fungi, with applications in medicine, agriculture, and environmental sciences.

## MATERIALS AND METHODS

### Microbial strains

The fungi *Alternaria alternata* Z4, *Nigrospora sphaerica* Z7, *Cyphellophora fusarioides,* and *Cladosporium colombiae* were isolated from the abdominal contents of the Antarctic fish *Trematomus bernacchii*, collected from the Antarctic China Zhongshan Station (69°22'27'' S, 76°22'20'' E), in February 2020. The terrestrial fungal isolates Q6 and N1 used in this study were isolated from dry air in Shanghai and taxonomically identified on a molecular basis as *Alternaria alternata* and *Nigrospora sphaerica*. Marine *A. alternata* K3, YC, and 23 were obtained from our laboratory and isolated from 6,900 and 7,332 m Mariana Trench sediments (11°20′ N, 142°11.5′ E, respectively). Terrestrial sources of *A. alternata* Q72 (CGMCC No. 3.6872) and *A. alternata* Z53 (CGMCC No. 3.17853) were obtained from Beijing air and plant rhizomes, respectively. All strains were grown at 28°C on potato dextrose agar (PDA), which consists of 200 g potato, 20 g glucose, 28 g NaCl, and 15 g agar.

### DNA extraction and phylogenetic analysis

Genomic DNA was extracted using the TIANcombi 145 DNA Lyse & Det PCR Kit (TIANGEN BIOTECH (BEIJING) CO., LTD). To confirm the identity of the fungal cultures, amplification was performed using primers ITS1/ITS4 (42) and Alt-for/Alt-rev (43), respectively, all of which are listed in Table S1. The PCR products were sequenced by Azenta Life Sciences Co., Ltd. Using the Sanger method. Sequence identity was confirmed by the NCBI blast (44), and the nucleotide sequences of the isolates were aligned with those of closely related strains using Mage (45) software to construct a phylogenetic tree.

### Co-culture and high-performance liquid chromatography (HPLC) analysis

To carry out the coculture, fungal strains were cultivated individually in 250 mL Erlenmeyer flasks containing 50 mL of PDB medium at 28°C on a rotary shaker at 120 rpm for 6 days. The mycelium was filtered through a sterile gauze to obtain spore suspensions (1 × 10^5^ cells/mL). Spores of both fungi were simultaneously inoculated into PDA plates (10 µL) or fresh rice solid medium (6 mL spore suspensions, 40 g rice, and 60 mL sterile seawater) and incubated at 28°C for 6 d or 30 d, respectively. The cocultures were extracted by an equivalent volume of ethyl acetate and dried *in vacuo*. The HPLC analysis was conducted with the Agilent 1260 Infinity II HPLC system using an Agilent Zorbax SB-C18 column (250 × 9.4 mm, 5 µm). The HPLC analytical condition was gradient elution in 5%–100% methanol (MeOH) in water for 50 min, washed with 100% (vol/vol) MeOH for 10 min. UV absorptions at 220 nm were illustrated.

### Mass coculture, extraction, and isolation of SMs

*A. alternata* Z4 and *N. sphaerica* Z7 were inoculated into 250 mL of PDB liquid medium and incubated at 28°C for 6 d on a rotary shaker (180 rpm). The supernatant and mycelia were separated by filtration. The supernatant (12 mL each) was transferred into 10 × 500 mL Erlenmeyer flask containing fresh rice solid medium at 28°C for 30 days. The rice solid culture was extracted with ethyl acetate three times and evaporated under reduced pressure to give a crude extract (1.08 g). Subsequently, the crude extract was fractionated by an octadecyl-functionalized silica gel column (ODS, Cosmosil 75C18-Prep) eluting with mixed solvents of MeOH/H_2_O (1:1, 7:3, 9:1, 1:0, vol/vol) to furnish four fractions (Fr-1–Fr-4) based on the results of HPLC analysis. Combine Fr. 2 and 3 (51.3 mg), and further purify using a gradient solvent system with 10%–100% MeOH in water containing 0.5% acetic acid (AcOH) over 50 min. Compound 1 (8.2 mg, t_R_ 45 min) was purified from Fr. 2-3 by SHIMADZU LC-20AP HPLC using a column (Agilent Zorbax SB-C18, 250 × 9.4 mm, 5 µm); the flow rate was set at 1 mL/min, with UV detection at 210 nm.

### NMR analysis

Nuclear magnetic resonance (NMR) spectra were recorded on a Bruker 500 spectrometer or a JEOL 600 spectrometer at room temperature. All spectra were processed with MestReNova 9.0, and the chemical shifts were expressed in δ (ppm) relative to DMSO-d6 (δC 39.5 and δH 2.50).

### LC-MS analysis

The liquid chromatograph mass spectrometer (LC-MS) analysis was performed on a Vanquish UPLC high-resolution mass spectrometer (Thermo Fisher), which was equipped with an electrospray ionization (ESI) source that operates in both positive and negative ion modes, using a Waters ACQUITY UPLC BEH C18 column (1.7 μm × 2.1 mm × 100 mm). The mobile phase consisted of water (A), and methanol (B) was used as the solvent at a flow rate of 0.4 mL/min. Calibration was performed based on the molecular weight and retention time of the AOH standard compound.

### Cytotoxic, antibacterial, and antifungal assay

The cytotoxic activities against human lung cancer NCI-H460, human liver cancer HepG2, human breast carcinoma MCF-7, and MDA-MB-231 cell lines were determined according to previously reported methods (46). The antibacterial and antifungal assay was conducted against *Salmonella choleraesuis*, *Mycobacterium smegmatis* MC2155, *Enterococcus faecalis* FA2-2, *Staphylococcus aureus* ATCC25923, and *Nigrospora sphaerica* Z7, by using the filter paper disk agar diffusion method (47). Six microliters (10 mg/mL) of the crude extract or compound was impregnated on sterile filter paper disks (6 mm diameter). MeOH or DMSO was employed as a negative control.

### RNA isolation

*A. alternata* Z4 and Q6 were cultivated in 50 mL of liquid PDB medium at 28°C, with or without *N. sphaerica* Z7. For the coculturing, *N. sphaerica* Z7 was sealed in a dialysis bag (molecular mass cutoff of 14 kDa), which was immersed in the flasks to avoid direct mycelial contact between the two species. After 3 d of incubation, the mycelium was ground to a fine powder using a mortar and pestle. The remaining mycelium and liquid were used for SM extraction and LC-MS analysis. Total RNA was extracted using the RNAiso Plus (TaKaRa, Dalian, China) reagent as described previously, and reverse transcription was performed using PrimeScript RT reagent kit (TaKaRa, Dalian, China). Glyceraldehyde-3-phosphate dehydrogenase (GAPDH) was selected as an internal reference gene for the calculation of expression levels. The oligonucleotide sequences for PCR primers are given in Table S1.

### Transcriptional analysis by RNA-seq and qRT-PCR

RNA sequencing was performed on a Novoseq X Plus (Illumina, San Diego, CA, USA) at the Novogene (Beijing, China). A total of three independent biological replicates were prepared under each condition. The clean data were trimmed from the raw reads for further analysis by removing reads containing adapters, reads containing N bases, and low-quality reads (Q value of ≤ 20). The read count data were subjected to differential expression (DE) analysis using the DESeq2 R package (1.20.0). The tool is based on a negative binomial distribution model, making it well suited for normalization and differential analysis of RNA-seq count data, with strong stability and a low false-positive rate. The false discovery rate (FDR) of <0.05 and the absolute value of a log2 fold change of ≥ 1 were used as the thresholds to judge the significance of the gene expression differences, ensuring the biological significance of the selected genes.

The qRT-PCR was carried out using the ABI 7500 Real Time PCR System (Thermo-Fisher) to assess the gene expression levels. The SYBR Premix Ex Taq II (Tli RNaseH Plus) (TaKaRa, Dalian, China) was used for the reactions. The qRT-PCR was performed in triplicate according to the previously reported protocol, and the relative expression levels were calculated using the 2^−∆∆Ct^ method (48). The qRT-PCR primers are listed in Table S1.

### Intracellular ROS levels and mitochondrial membrane potential assay

2,7-dichlorodihydrofluorescein diacetate (DCFH-DA) (49) is a widely used fluorescent probe for the detection of intracellular ROS. Once inside the cell, it is deacetylated by esterases to yield non-fluorescent DCFH, which can then be oxidized by ROS to produce the highly fluorescent compound DCF, thereby serving as an indicator of ROS levels. Therefore, basal levels of ROS, even in the absence of external stimuli or co-cultivation, can generate detectable fluorescence. JC-1 (5,5′,6,6′-tetrachloro-1,1′,3,3′-tetraethyl-imidacarbocyanine) (50) is a mitochondrial membrane potential probe that forms red-fluorescent aggregates at high membrane potentials, while existing as green fluorescent monomers at low membrane potentials, thus providing an effective means to evaluate mitochondrial functional status. The spore suspension (1 × 10^5^ cells/mL) was inoculated into the PDB medium at a 1% inoculation rate. After 3 days of cocultivation, mature mycelia were harvested, washed twice with PBS, and then resuspended in 10 µM DCFH-DA and JC-1 solutions according to the instructions (Beyotime Biotech, Shanghai, China), respectively. The suspensions were then stained in the dark at 37°C for 4 h or 30 min to detect intracellular ROS and mitochondrial membrane potential levels. After washing with PBS, the mycelia were observed by epifluorescence microscopy (Olympus U-LH100HG, Tokyo, Japan; 40 × magniﬁcation). All staining experiments and groups were conducted in triplicate.

To quantify specific intracellular ROS values, stained *A. alternata* mycelium was washed with PBS, ground to a fine powder with liquid nitrogen, and then mixed with 1 mL of cold PBS buffer; 200 mL of the supernatant was measured using an Infinite M PLEX (Tecan Trading AG, Switzerland) with excitation and emission wavelengths of 488 nm and 525 nm, respectively.

### Application of antioxidants in coculture

To remove ROS generated in *A. alternata* Z4 during coculture with *N. sphaerica* Z7, ROS scavenger N-acetyl-L-cysteine (NAC, Sigma, St. Louis, MO, USA) was used. At the beginning of the coculture, NAC was added to 50 mL of the PDB medium to reach a final concentration of 1 mM. *N. sphaerica* Z7 was separated using dialysis bags. After 3 days of incubation, the mycelia were collected by filtering through a sterile gauze, ground in liquid nitrogen, and ROS levels were measured. The remaining mycelia and liquid were extracted with ethyl acetate to obtain crude extracts. These crude extracts were analyzed by HPLC together with AOH standards to determine the peak area of AOH in the extracts.

## Data Availability

Transcriptomic data are publicly available in National Center for Biotechnology Information (NCBI) under BioProject number PRJNA1127049. All relevant data are within the paper and its Supporting Information files.

## SUPPLEMENTAL MATERIAL

Supplemental material mbio.01447-25-s0001.docx Fig. S1 to S13; Tables S1 to S4.

ASM does not own the copyrights to Supplemental Material that may be linked to, or accessed through, an article. The authors have granted ASM a non-exclusive, world-wide license to publish the Supplemental Material files. Please contact the corresponding author directly for reuse.

## References

[1] Gladfelter AS, James TY, Amend AS. 2019. Marine fungi. Curr Biol 29:R191–R195. doi:10.1016/j.cub.2019.02.009 30889385

[2] Cunliffe M. 2023. Who are the marine fungi? Environ Microbiol 25:131–134. doi:10.1111/1462-2920.16240 36217720 PMC10092172

[3] Macheleidt J, Mattern DJ, Fischer J, Netzker T, Weber J, Schroeckh V, Valiante V, Brakhage AA. 2016. Regulation and role of fungal secondary metabolites. Annu Rev Genet 50:371–392. doi:10.1146/annurev-genet-120215-035203 27732794

[4] Wang G, Ran H, Fan J, Keller NP, Liu Z, Wu F, Yin WB. 2022. Fungal-fungal cocultivation leads to widespread secondary metabolite alteration requiring the partial loss-of-function VeA1 protein. Sci Adv 8:eabo6094. doi:10.1126/sciadv.abo6094 35476435 PMC9045611

[5] Lee N, Kim W, Chung J, Cho S, Jang KS, Palsson B, Cho BK. 2018. Iron competition triggers antibiotic biosynthesis in Streptomyces coelicolor during coculture with Myxococcus xanthus. ISME J 14:1111–1124. doi:10.1038/s41396-020-0594-6 PMC7174319 31992858

[6] Čihák M, Kameník Z, Šmídová K, Bergman N, Benada O, Kofroňová O, Petříčková K, Bobek J. 2017. Secondary metabolites produced during the germination of Streptomyces coelicolor. Front Microbiol 8:2495. doi:10.3389/fmicb.2017.02495 29326665 PMC5733532

[7] Xu S, Li M, Hu Z, Shao Y, Ying J, Zhang H. 2023. The potential use of fungal co-culture strategy for discovery of new secondary metabolites. Microorganisms 11:464. doi:10.3390/microorganisms11020464 36838429 PMC9965835

[8] Bhalkar BN, Patil SM, Govindwar SP. 2016. Camptothecine production by mixed fermentation of two endophytic fungi from Nothapodytes nimmoniana. Fungal Biol 120:873–883. doi:10.1016/j.funbio.2016.04.003 27268247

[9] Chagas FO, Dias LG, Pupo MT. 2013. A mixed culture of endophytic fungi increases production of antifungal polyketides. J Chem Ecol 39:1335–1342. doi:10.1007/s10886-013-0351-7 24114180

[10] Li F, Yan S, Huang Z, Gao W, Zhang S, Mo S, Lin S, Wang J, Hu Z, Zhang Y. 2021. Inducing new bioactive metabolites production from coculture of Pestalotiopsis sp. and Penicillium bialowiezense. Bioorg Chem 110:104826. doi:10.1016/j.bioorg.2021.104826 33780746

[11] Ogawa M, García-Martínez T, Bisson L, Mauricio JC, Moreno J, Moreno-García J. 2020. Mapping the intracellular metabolome of yeast biocapsules - spherical structures of yeast attached to fungal pellets. N Biotechnol 58:55–60. doi:10.1016/j.nbt.2020.05.003 32562862

[12] Yaakoub H, Mina S, Calenda A, Bouchara JP, Papon N. 2022. Oxidative stress response pathways in fungi. Cell Mol Life Sci 79:333. doi:10.1007/s00018-022-04353-8 35648225 PMC11071803

[13] Singh Y, Nair AM, Verma PK. 2021. Surviving the odds: from perception to survival of fungal phytopathogens under host-generated oxidative burst. Plant Commun 2:100142. doi:10.1016/j.xplc.2021.100142 34027389 PMC8132124

[14] Yang Q, Yang J, Wang Y, Du J, Zhang J, Luisi BF, Liang W. 2022. Broad-spectrum chemicals block ROS detoxification to prevent plant fungal invasion. Curr Biol 32:3886–3897. doi:10.1016/j.cub.2022.07.022 35932761 PMC7613639

[15] Steenwyk JL. 2021. Evolutionary divergence in DNA damage responses among fungi. mBio 12:e03348-20. doi:10.1128/mBio.03348-20 33727357 PMC8092291

[16] Li J, Xiao X, Zhou M, Zhang Y. 2023. Strategy for the adaptation to stressful conditions of the novel isolated conditional piezophilic strain Halomonas titanicae ANRCS81. Appl Environ Microbiol 89:e0130422. doi:10.1128/aem.01304-22 36912687 PMC10057041

[17] Nanda AK, Andrio E, Marino D, Pauly N, Dunand C. 2010. Reactive oxygen species during plant-microorganism early interactions. J Integr Plant Biol 52:195–204. doi:10.1111/j.1744-7909.2010.00933.x 20377681

[18] Heller J, Tudzynski P. 2011. Reactive oxygen species in phytopathogenic fungi: signaling, development, and disease. Annu Rev Phytopathol 49:369–390. doi:10.1146/annurev-phyto-072910-095355 21568704

[19] Fountain JC, Bajaj P, Nayak SN, Yang L, Pandey MK, Kumar V, Jayale AS, Chitikineni A, Lee RD, Kemerait RC, Varshney RK, Guo B. 2016. Responses of Aspergillus flavus to oxidative stress are related to fungal development regulator, antioxidant enzyme, and secondary metabolite biosynthetic gene expression. Front Microbiol 7:2048. doi:10.3389/fmicb.2016.02048 28066369 PMC5175028

[20] Shabana S, Lakshmi KR, Satya AK. 2021. An updated review of secondary metabolites from marine fungi. Mini Rev Med Chem 21:602–642. doi:10.2174/1389557520666200925142514 32981503

[21] Deshmukh SK, Prakash V, Ranjan N. 2017. Marine fungi: a source of potential anticancer compounds. Front Microbiol 8:2536. doi:10.3389/fmicb.2017.02536 29354097 PMC5760561

[22] Shaker S, Sun TT, Wang LY, Ma WZ, Wu DL, Guo YW, Dong J, Chen YX, Zhu LP, Yang DP, Li HJ, Lan WJ. 2021. Reactive oxygen species altering the metabolite profile of the marine-derived fungus Dichotomomyces cejpii F31-1. Nat Prod Res 35:41–48. doi:10.1080/14786419.2019.1611816 31215239

[23] Aly AH, Edrada-Ebel R, Indriani ID, Wray V, Müller WEG, Totzke F, Zirrgiebel U, Schächtele C, Kubbutat MHG, Lin WH, Proksch P, Ebel R. 2008. Cytotoxic metabolites from the fungal endophyte Alternaria sp. and their subsequent detection in its host plant Polygonum senegalense. J Nat Prod 71:972–980. doi:10.1021/np070447m 18494522

[24] Gutierrez RMP, Gonzalez AMN, Ramirez AM. 2012. Compounds derived from endophytes: a review of phytochemistry and pharmacology. Curr Med Chem 19:2992–3030. doi:10.2174/092986712800672111 22489725

[25] Wenderoth M, Garganese F, Schmidt‐Heydt M, Soukup ST, Ippolito A, Sanzani SM, Fischer R. 2019. Alternariol as virulence and colonization factor of Alternaria alternata during plant infection. Mol Microbiol 112:131–146. doi:10.1111/mmi.14258 30947377

[26] Yu W, Pei R, Zhou J, Zeng B, Tu Y, He B. 2023. Molecular regulation of fungal secondary metabolism. World J Microbiol Biotechnol 39:204. doi:10.1007/s11274-023-03649-6 37209190

[27] Ryan DG, Frezza C, O’Neill LA. 2021. TCA cycle signalling and the evolution of eukaryotes. Curr Opin Biotechnol 68:72–88. doi:10.1016/j.copbio.2020.09.014 33137653 PMC7116391

[28] Zhang X, Wang Z, Jiang C, Xu JR. 2021. Regulation of biotic interactions and responses to abiotic stresses by MAP kinase pathways in plant pathogenic fungi. Stress Biol 1:5. doi:10.1007/s44154-021-00004-3 37676417 PMC10429497

[29] Schink SJ, Christodoulou D, Mukherjee A, Athaide E, Brunner V, Fuhrer T, Bradshaw GA, Sauer U, Basan M. 2022. Glycolysis/gluconeogenesis specialization in microbes is driven by biochemical constraints of flux sensing. Mol Syst Biol 18. doi:10.15252/msb.202110704 PMC8738977 34994048

[30] McCarthy MW, Walsh TJ. 2018. Amino acid metabolism and transport mechanisms as potential antifungal targets. Int J Mol Sci 19:909. doi:10.3390/ijms19030909 29562716 PMC5877770

[31] Simaan H, Lev S, Horwitz BA. 2019. Oxidant-sensing pathways in the responses of fungal pathogens to chemical stress signals. Front Microbiol 10:567. doi:10.3389/fmicb.2019.00567 30941117 PMC6433817

[32] Kemp MG. 2017. DNA damage-induced ATM- and Rad-3-related (ATR) kinase activation in non-replicating cells is regulated by the XPB subunit of transcription factor IIH (TFIIH). J Biol Chem 292:12424–12435. doi:10.1074/jbc.M117.788406 28592488 PMC5535018

[33] Alonso-Monge R, Navarro-García F, Román E, Negredo AI, Eisman B, Nombela C, Pla J. 2003. The Hog1 mitogen-activated protein kinase is essential in the oxidative stress response and chlamydospore formation in Candida albicans. Eukaryot Cell 2:351–361. doi:10.1128/EC.2.2.351-361.2003 12684384 PMC154845

[34] Bavarsad Shahripour R, Harrigan MR, Alexandrov AV. 2014. N-acetylcysteine (NAC) in neurological disorders: mechanisms of action and therapeutic opportunities. Brain Behav 4:108–122. doi:10.1002/brb3.208 24683506 PMC3967529

[35] Wang L, Wang M, Jiao J, Liu H. 2022. Roles of AaVeA on mycotoxin production via light in alternaria alternata. Front Microbiol 13:842268. doi:10.3389/fmicb.2022.842268 35250954 PMC8894881

[36] Pruss S, Fetzner R, Seither K, Herr A, Pfeiffer E, Metzler M, Lawrence CB, Fischer R. 2014. Role of the Alternaria alternata blue-light receptor LreA (white-collar 1) in spore formation and secondary metabolism. Appl Environ Microbiol 80:2582–2591. doi:10.1128/AEM.00327-14 24532063 PMC3993185

[37] Oviedo MS, Ramirez ML, Barros GG, Chulze SN. 2010. Impact of water activity and temperature on growth and alternariol and alternariol monomethyl ether production of Alternaria alternata isolated from soybean. J Food Prot 73:336–343. doi:10.4315/0362-028x-73.2.336 20132680

[38] Brzonkalik K, Hümmer D, Syldatk C, Neumann A. 2012. Influence of pH and carbon to nitrogen ratio on mycotoxin production by Alternaria alternata in submerged cultivation. AMB Express 2:28. doi:10.1186/2191-0855-2-28 22608165 PMC3441619

[39] Brzonkalik K, Herrling T, Syldatk C, Neumann A. 2011. Process development for the elucidation of mycotoxin formation in Alternaria alternata. AMB Express 1:27. doi:10.1186/2191-0855-1-27 21970547 PMC3222323

[40] Harrison KA, Bol R, Bardgett RD. 2008. Do plant species with different growth strategies vary in their ability to compete with soil microbes for chemical forms of nitrogen? Soil Biol Biochem 40:228–237. doi:10.1016/j.soilbio.2007.08.004

[41] Liu J, Peng C, Han Q, Wang M, Zhou G, Ye B, Xiao Y, Fang Z, Kües U. 2022. Coprinopsis cinerea uses laccase Lcc9 as a defense strategy to eliminate oxidative stress during fungal‐fungal interactions. Appl Environ Microbiol 88:e0176021. doi:10.1128/AEM.01760-21 34669425 PMC8752157

[42] Pryor BM, Gilbertson RL. 2000. Molecular phylogenetic relationships amongst Alternaria species and related fungi based upon analysis of nuclear ITS and mt SSU rDNA sequences. Mycol Res 104:1312–1321. doi:10.1017/S0953756200003002

[43] Hong SG, Cramer RA, Lawrence CB, Pryor BM. 2005. Alt a 1 allergen homologs from Alternaria and related taxa: analysis of phylogenetic content and secondary structure. Fungal Genet Biol 42:119–129. doi:10.1016/j.fgb.2004.10.009 15670710

[44] Sayers EW, Bolton EE, Brister JR, Canese K, Chan J, Comeau DC, Connor R, Funk K, Kelly C, Kim S, Madej T, Marchler-Bauer A, Lanczycki C, Lathrop S, Lu Z, Thibaud-Nissen F, Murphy T, Phan L, Skripchenko Y, Tse T, Wang J, Williams R, Trawick BW, Pruitt KD, Sherry ST. 2022. Database resources of the national center for biotechnology information. Nucleic Acids Res 50:D20–D26. doi:10.1093/nar/gkab1112 34850941 PMC8728269

[45] Kumar S, Nei M, Dudley J, Tamura K. 2008. MEGA: a biologist-centric software for evolutionary analysis of DNA and protein sequences. Brief Bioinformatics 9:299–306. doi:10.1093/bib/bbn017 18417537 PMC2562624

[46] Xiao Y, Yan F, Cui Y, Du J, Hu G, Zhai W, Liu R, Zhang Z, Fang J, Chen L, Yu X. 2022. A symbiotic bacterium of Antarctic fish reveals environmental adaptability mechanisms and biosynthetic potential towards antibacterial and cytotoxic activities. Front Microbiol 13:1085063. doi:10.3389/fmicb.2022.1085063 36713225 PMC9882997

[47] Zeng YB, Wang H, Zuo WJ, Zheng B, Yang T, Dai HF, Mei WL. 2012. A fatty acid glycoside from a marine-derived fungus isolated from mangrove plant Scyphiphora hydrophyllacea. Mar Drugs 10:598–603. doi:10.3390/md10030598 22611356 PMC3347017

[48] Livak KJ, Schmittgen TD. 2001. Analysis of relative gene expression data using real-time quantitative PCR and the 2^−ΔΔC_T_^ method. Methods 25:402–408. doi:10.1006/meth.2001.1262 11846609

[49] Narasaiah KV, Sashidhar RB, Subramanyam C. 2006. Biochemical analysis of oxidative stress in the production of aflatoxin and its precursor intermediates. Mycopathologia 162:179–189. doi:10.1007/s11046-006-0052-7 16944285

[50] Maryam A, Mehmood T, Yan Q, Li Y, Khan M, Ma T. 2018. Proscillaridin a promotes oxidative stress and ER stress, inhibits STAT3 activation, and induces apoptosis in A549 lung adenocarcinoma cells. Oxid Med Cell Longev 2018:3853409. doi:10.1155/2018/3853409 29576846 PMC5821950

