# Supplementary material for: Fungal-fungal cocultivation alters secondary metabolites of marine fungi mediated by reactive oxygen species (ROS)
Source: mBio. 2025 Aug 18;16(9):e01447-25. doi: 10.1128/mbio.01447-25 (PMC12421890; doi:10.1128/mbio.01447-25)
Supplement: Supplemental material — Fig. S1 to S13; Tables S1 to S4. [file mbio.01447-25-s0001.docx]

**Fungal-fungal cocultivation alters secondary metabolites of marine fungi mediated by reactive oxygen species (ROS)**

Yu Xiao^1^, Yongqi Li^1^, Yukun Cui^1^, Paiyao Ji^1^, Zhizhen Zhang^2^, Jiasong Fang^1^ and Xi Yu^1*^

**running head**: ROS alters metabolites in marine fungal cocultivation

**Address**: ^1^Shanghai Engineering Research Center of Hadal Science and Technology, College of Oceanography and Ecological Science, Shanghai Ocean University, Shanghai, 201306, China

^2^Ocean College, Zhoushan Campus, Zhejiang University, Zhoushan, 316021, China

*corresponding author: XY (x[yu@shou.edu.cn](mailto:yu@shou.edu.cn));

**Key words:** fungi, cocultivation, gene clusters, secondary metabolites, oxidative stress

**Figure S1**


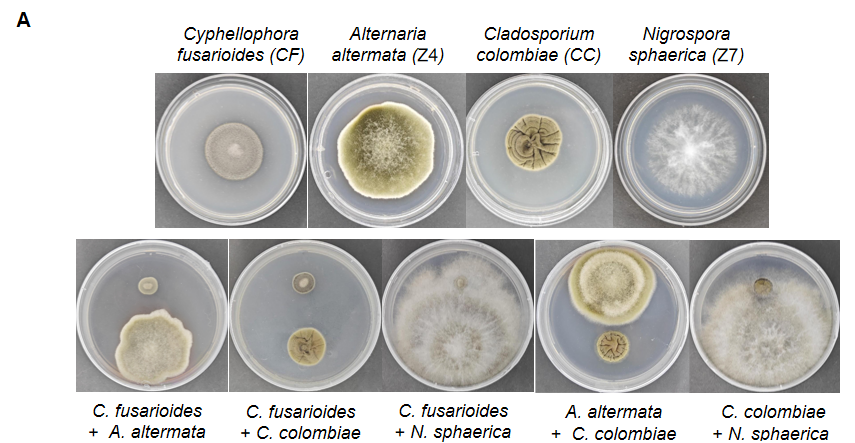


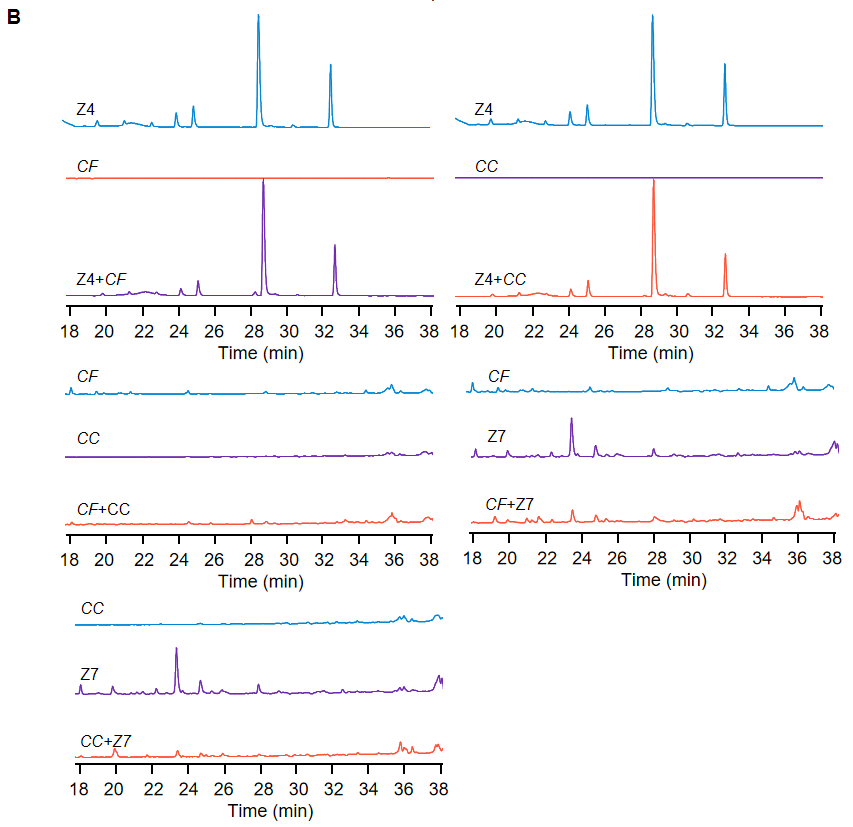


**Figure S1. Effects of coculture of marine fungi on secondary metabolites. (A)** Growth of marine fungi on PDA plates under monoculture and coculture conditions. **(B)** HPLC analysis of crude extracts from monocultivated and cocultivated strains. Ultraviolet (UV) absorptions at 220 nm are illustrated. CF, *C. fusarioides*; Z4, *A altermata*; CC, *C. colombiae*; Z7, *N. sphaerica*.

**Figure S2**


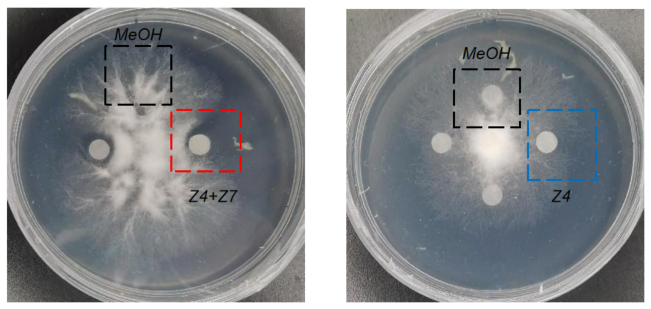


**Figure S2. The antimicrobial activity of crude extracts of *A. alternata* Z4.** Inhibition zone of *N. sphaerica* Z7 by crude extracts of *A. alternata* Z4 monocultures and cocultures. *Red box* *A. alternata* Z4+*N. sphaerica* Z7 cocultivation; *blue box* *A. alternata* Z4; *black box* MeOH.

**Figure S3**


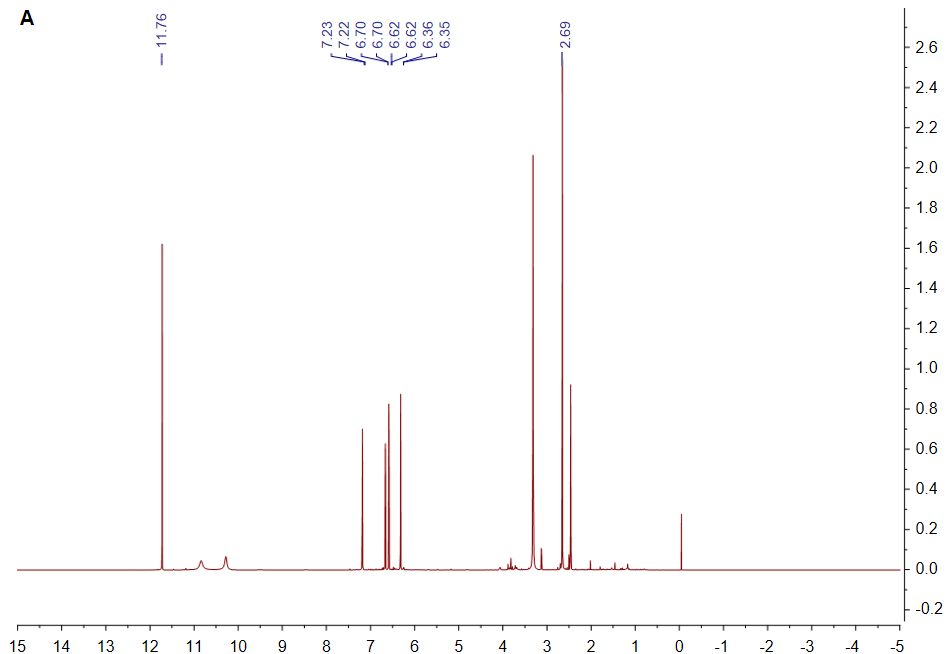

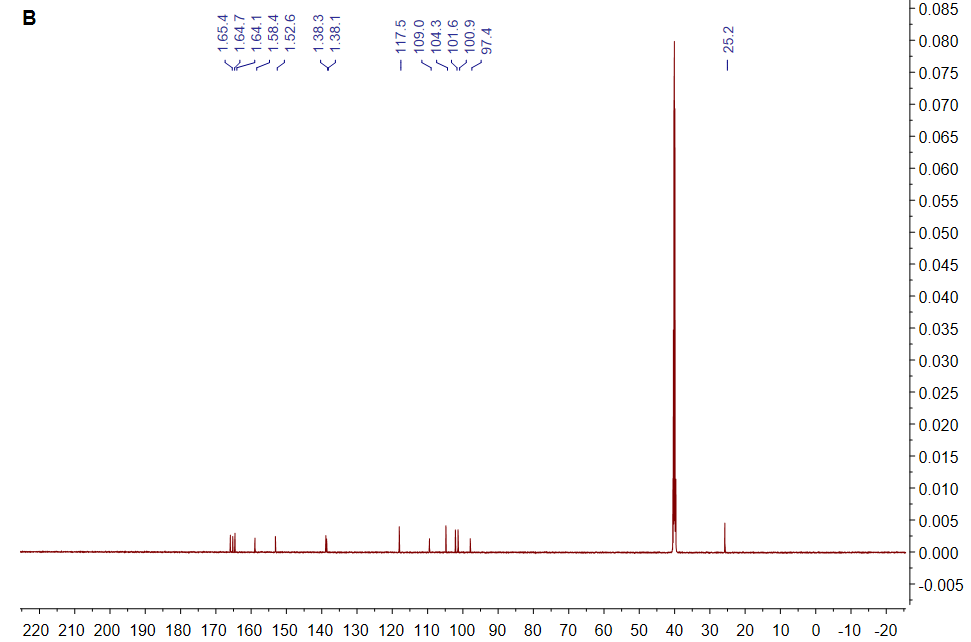


**Figure S3. Identification of the bioactive compound alternariol. (A)** ^1^H-NMR spectrum of alternariol in DMSO-*d*_6_. **(B)** ^13^C-NMR spectrum of alternariol in DMSO-*d*_6_.

**Figure S4**


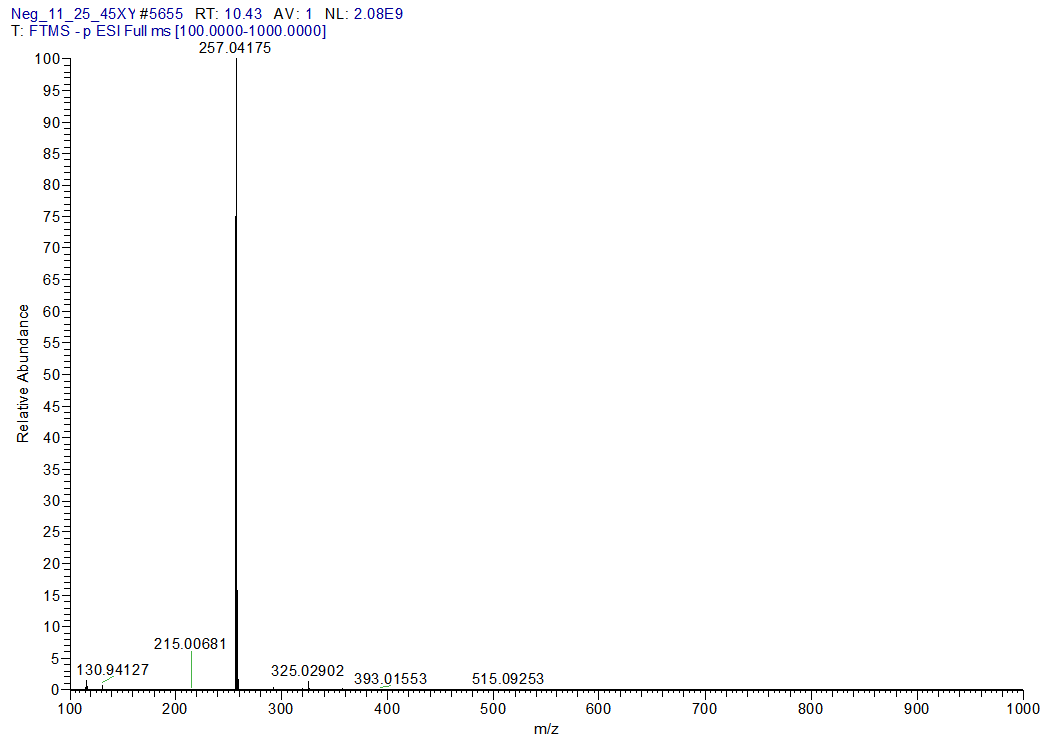


**Figure S4. LC-MS analysis of alternariol.** Total ion chromatogram (TIC) is illustrated in negative ion mode, *m/z* 257 [M-H].

**Figure S5**

**
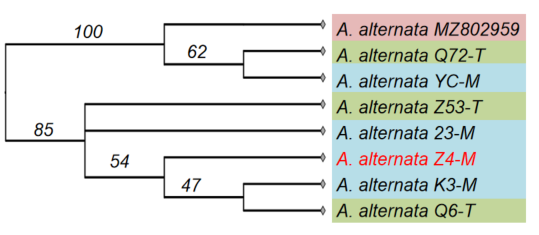
**

**Figure S5. Identification in *A. alternata* from different sources.** Phylogenetic tree based on Alt gene sequences of six *A. alternata*. The numbers at the nodes indicate the bootstrap values. *Red box*, reference strain; *green box*, terrestrial strain; *blue box*, marine strain. itol.embl.de.

**Figure S6**


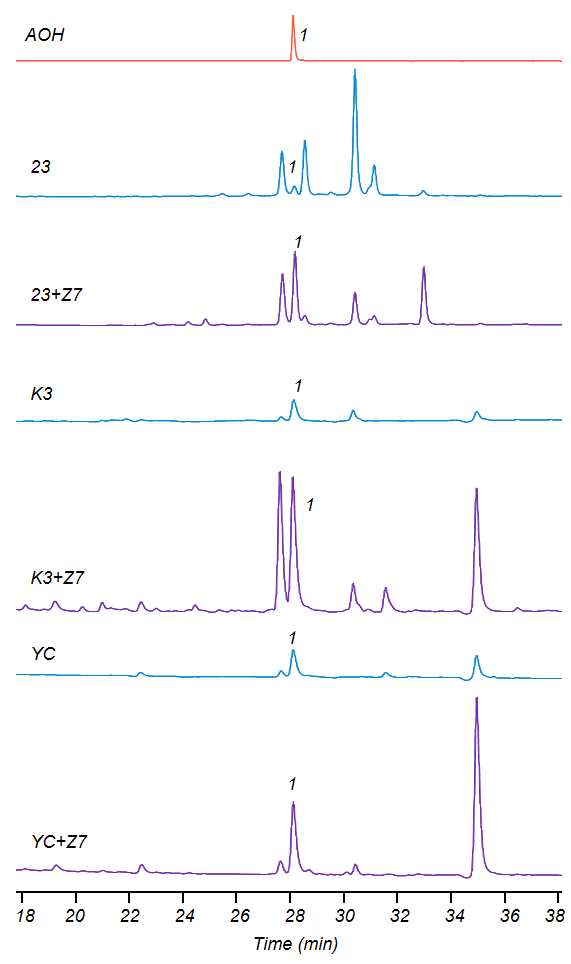


**Figure S6. HPLC analysis of crude extracts from monocultivated and cocultivated strains.** 23, *A. alternata* 23; 23+Z7, *A. alternata* 23+*N. sphaerica* Z7 cocultivation; K3, *A. alternata* K3; K3+Z7, *A. alternata* K3+*N. sphaerica* Z7 cocultivation; YC, *A. alternata* YC; YC+Z7, *A. alternata* YC+*N. sphaerica* Z7 cocultivation.

**Figure S7**


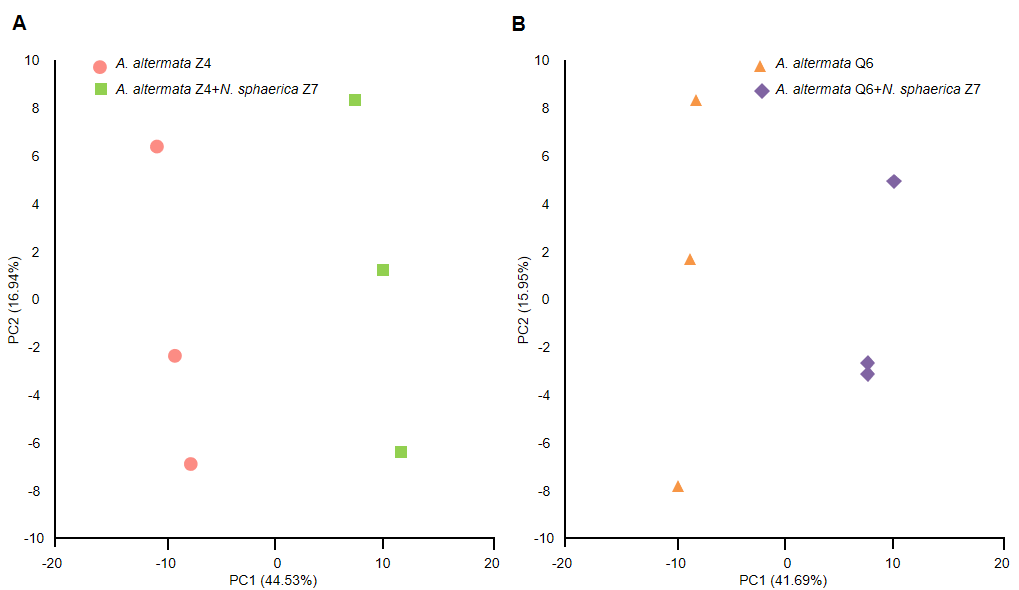


**Figure S7. Principal component analysis (PCA) plot based on the transcriptomic data. (A)** Mono-cultivation of *A. alternata* Z4 are in red; and cocultivation of *A. alternata* Z4 with *N. sphaerica* Z7 are in green. **(B)** Mono-cultivation of *A. alternata* Q6 are in orange; and cocultivation of *A. alternata* Q6 with *N. sphaerica* Z7 are in violet. Three biological replicates were analyzed for each group.

**Figure S8**


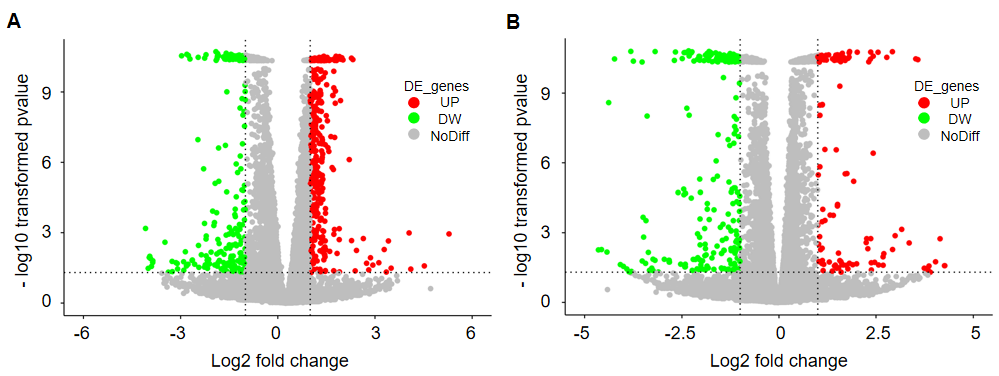


**Figure S8. The volcano map of differentially expressed genes. (A)** Volcanic mapping of differentially expressed genes in *A. alternata* Z4 coculture. **(B)** Volcanic mapping of differentially expressed genes in *A. alternata* Q6 coculture. The up-regulated differentially expressed genes are in red; down-regulated differentially expressed genes are in green. and non-significantly differentially expressed genes are in grey.

**Figure S9**


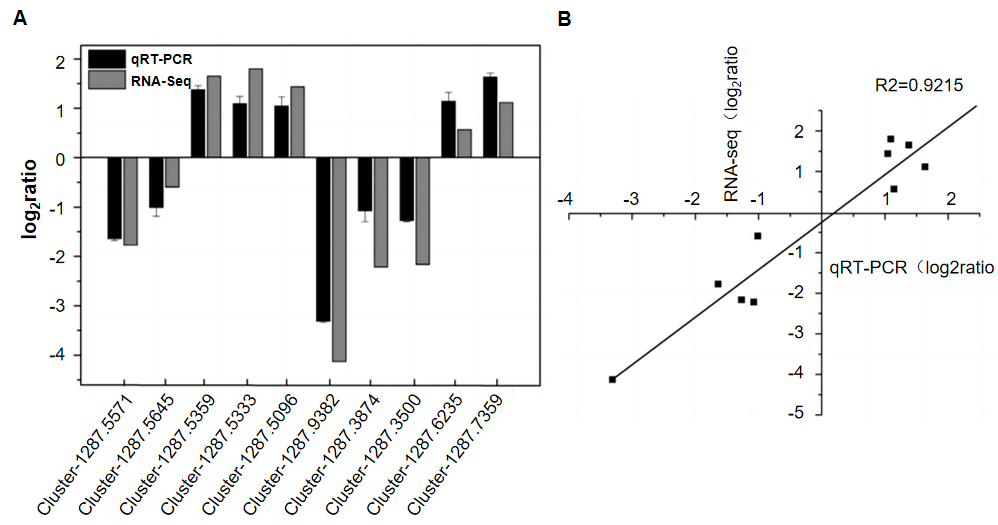


**Figure S9. Expression levels of differently expressed genes validated by RT-qPCR. (A)** 10 genes were selected to verify the results of RNA-Seq by RT-qPCR. The five genes on the left were selected from *A. alternata* Z4 coculture and the five genes on the right were selected from *A. alternata* Q6 coculture. **(B)** The value correlations between RNA-seq and RT-qPCR. R^2^ indicates the R-square of the regression line. The correlations between RNA-Seq and RT-qPCR exhibited well (R^2^ > 0.9).

**Figure S10**


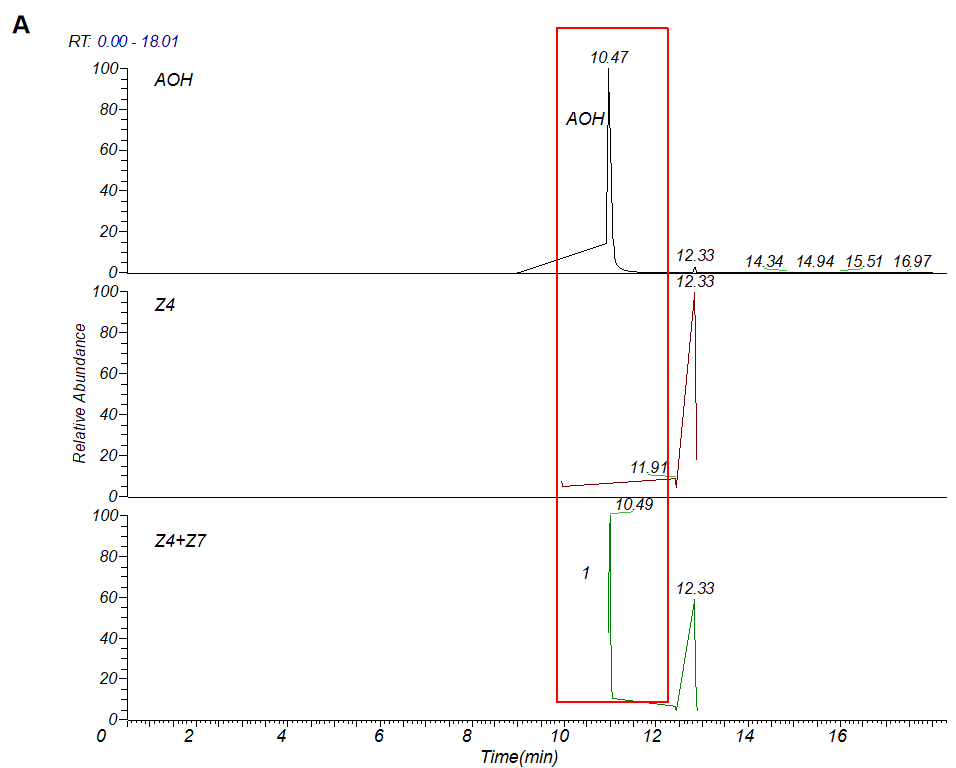


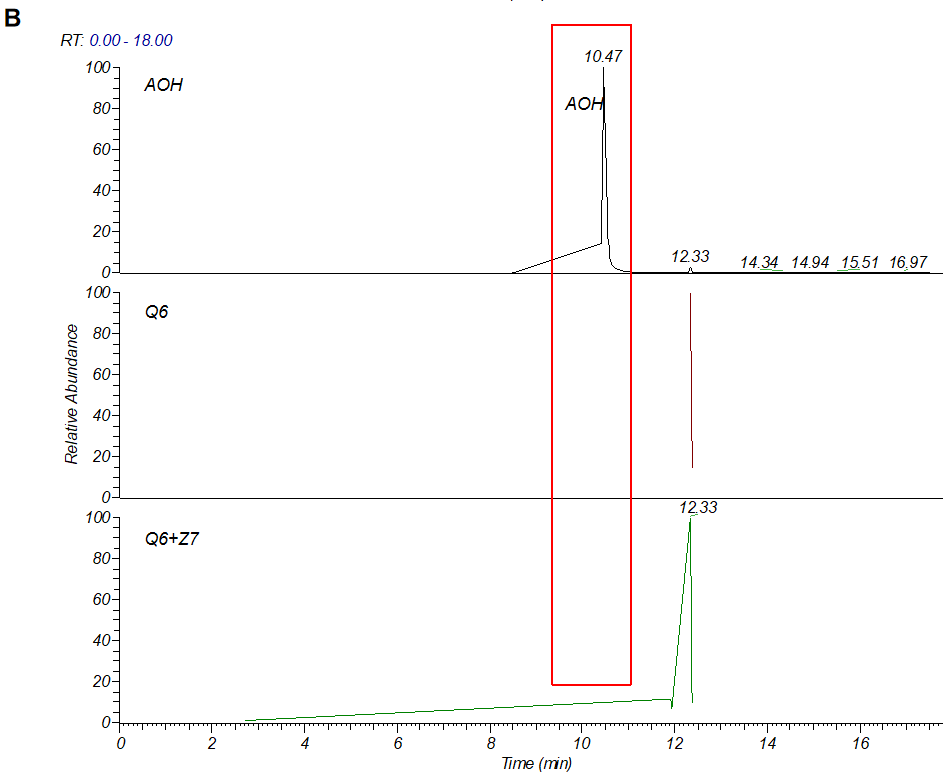


**Figure S10. LC-MS analysis of *A. alternata* coculture extracts with *N. sphaerica* Z7 strains.** **(A)** Secondary mass spectra of cocultured extracts of *A. alternata* Z4 and *N. sphaerica* Z7. **(B)** Secondary mass spectra of cocultured extracts of *A. alternata* Q6 and *N. sphaerica* Z7. AOH, alternariol.

**Figure S11**


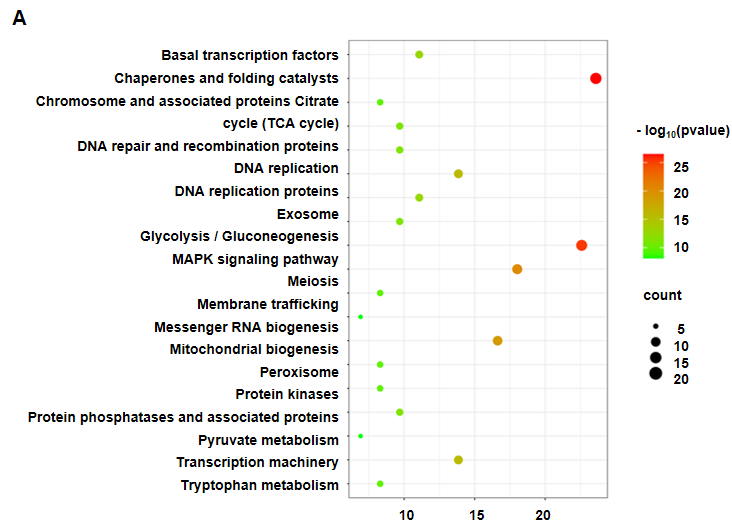

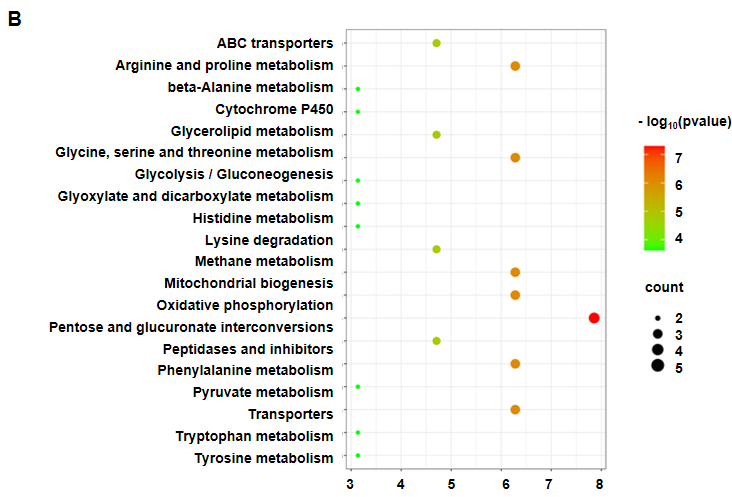


**Figure S11. KEGG enrichment analysis of differentially expressed genes in coculture condition. (A)** *A. alternata* Z4 coculture vs *A. alternata* Z4. **(B)** *A. alternata* Q6 coculture vs *A. alternata* Q6 coculture.

**Figure S12**


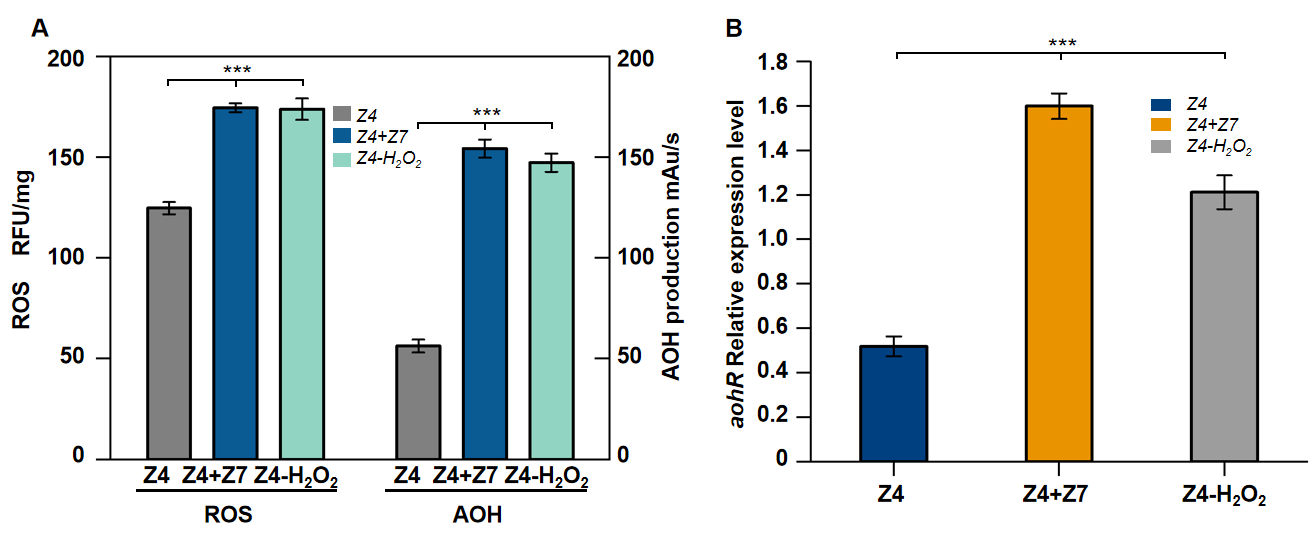


**Figure S12. Characteristics of *A. alternata* Z4 in response to H2O2 stress.** (A) ROS level and AOH production level of *A. alternata* Z4 in different culture conditions. (B) Relative expression level of *aohR* of *A. alternata* Z4 in different culture conditions. The results were expressed as mean ± standard deviation (SD). The experiments were repeated tree times with similar results. The statistical significance of the fluorescence intensity of ROS, the peak area of alternariol and *aohR* gene expression level compared with the control group was determined using a two-tailed Student′st test; *** represents P <0.0001. Plotted with Origin 2022.

**Figure S13**


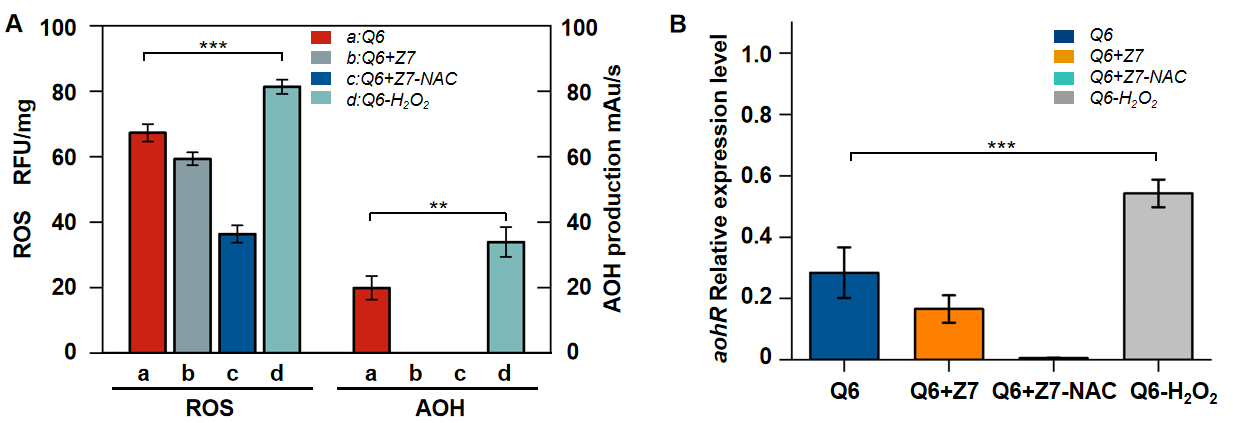


**Figure S13. Characterisation of the response of *A. alternata* Q6 to different culture conditions.** (A) ROS level and AOH production level of *A. alternata* Q6 in different culture conditions. (B) Relative expression level of *aohR* of *A. alternata* Q6 in different culture conditions. The results were expressed as mean ± standard deviation (SD). The experiments were repeated tree times with similar results. The statistical significance of the fluorescence intensity of ROS, the peak area of alternariol and *aohR* gene expression level compared with the control group was determined using a two-tailed Student′st test, * represents *P* <0.05, ** represents *P* <0.01, *** represents *P* <0.0001. Plotted with Origin 2022.

**Table S1. Primers in this study.**

| **Primer name** | **Primer sequence 5’ – 3’** | **Annealing temperature** | **Purpose** |
| --- | --- | --- | --- |
| ITS1 | TCCGTAGGTGAACCTGCGG | 55 | phylogeny marker |
| ITS4 | TCCTCCGCTTATTGATATGC |  |  |
| Alt-for | ATGCAGTTCACCACCATCGC | 65 | phylogeny marker |
| Alt-rev | ACGAGGGTGAYGTAGGCGTC |  |  |
| GADPH-for | GGCAAGACCATCCGTTTC | 55 | qPCR |
| GADPH-rev | CAGCAGAGGGAGCAGAAAT |  |  |
| Cluster-1287.5571-for | CCTGGCGGGTGTTGATAC | 55 | qPCR |
| Cluster-1287.5571-rev | CATGCACGACCACTGGAA |  |  |
| Cluster-1287.5645-for | ATGGAAGAAGCACGAAGC | 55 | qPCR |
| Cluster-1287.5645-rev | CGCCGTTAGACAAAGAGT |  |  |
| Cluster-1287.5359-for | GCGGCGACAAGAAGATAG | 55 | qPCR |
| Cluster-1287.5359-rev | GTTTCGGACGTAAGGGTG |  |  |
| Cluster-1287.5333-for | CTTTGCCGCCTTTGTCTT | 55 | qPCR |
| Cluster-1287.5333-rev | CTGATTGCTGCCAACTCTA |  |  |
| Cluster-1287.5096-for | CTGCTTGCTATTGAGGGAC | 55 | qPCR |
| Cluster-1287.5096-rev | TTCTTGCGGAGATGGTTC |  |  |
| Cluster-1287.9382-for | TTTCCGATGCTGGTGATG | 55 | qPCR |
| Cluster-1287.9382-rev | TTTGCGTAGACCTGGGTG |  |  |
| Cluster-1287.3874-for | GGGGTAGACAGCGATGAG | 55 | qPCR |
| Cluster-1287.3874-rev | CGACTTGGAGGCTATTGG |  |  |
| Cluster-1287.3500-for | GTTTATGCGGGCTGCTCT | 55 | qPCR |
| Cluster-1287.3500-rev | CTGCGTCGGATTCTGTGC |  |  |
| Cluster-1287.6235-for | AGACCGACACCATAACCG | 55 | qPCR |
| Cluster-1287.6235-rev | GAGCCAGACCTCAGCATC |  |  |
| Cluster-1287.7359-for | CCTACGACGGTCAAGTGC | 55 | qPCR |
| Cluster-1287.7359-rev | ACCAGAAACGGTGCCAAT |  |  |
| *aohR*-for | CAGAAGTCAAAGTCCAGGAT | 55 | qPCR |
| *aohR*-rev | TCTTGCATCTATCCACCAAG |  |  |

**Table S2. NMR Data of alternariol at 600 (^1^H) and 150 (^13^C) MHz (in DMSO-*d*_6_).**

| **position** | **δH mult. (J Hz)** | **δC** |
| --- | --- | --- |
| 1 |  | 138.1 |
| 2 |  | 97.4 |
| 3 |  | 164.7 |
| 4 | 6.36 d (2.1) | 100.9 |
| 5 |  | 165.4 |
| 6 | 7.22 d (2.1) | 104.3 |
| 7 |  | 164.1 |
| 1’ |  | 109 |
| 2’ |  | 152.6 |
| 3’ | 6.62 d (2.6) | 101.6 |
| 4’ |  | 158.4 |
| 5’ | 6.70 d (2.6) | 117.5 |
| 6’ |  | 138.3 |
| CH3 | 2.69 s | 25.2 |
| OH | 11.76 s |  |

**Table S3. Statistical summary of the results of RNA-seq data.** Z4 represents *A. alternata* Z4 monoculture; Z47 represents *A. alternata* Z4 and *N. sphaerica* Z7 coculture; Q6 represents *A. alternata* Q6 monoculture; Q67 represents *A. alternata* Q6 and *N. sphaerica* Z7 coculture.

| **sample** | **raw_reads** | **raw_bases** | **clean_reads** | **clean_bases** | **Q20(%)** | **Q30(%)** | **clean read ratio (%)** |
| --- | --- | --- | --- | --- | --- | --- | --- |
| Z4_1 | 23,286,960 | 6.99 | 20,920,199 | 6.28 | 98.42 | 95.52 | 89.84 |
| Z4_2 | 23,511,896 | 7.05 | 22,110,513 | 6.63 | 98.31 | 95.27 | 94.04 |
| Z4_3 | 23,489,767 | 7.05 | 21,955,023 | 6.59 | 98.42 | 95.5 | 93.47 |
| Z4+Z7_1 | 23,393,533 | 7.02 | 21,752,203 | 6.53 | 98.29 | 95.2 | 92.98 |
| Z4+Z7_2 | 27,269,597 | 8.18 | 25,464,385 | 7.64 | 98.14 | 94.9 | 93.38 |
| Z4+Z7_3 | 23,310,912 | 6.99 | 21,640,908 | 6.49 | 98.25 | 95.1 | 92.84 |
| Q6_1 | 23,980,725 | 7.19 | 22,342,588 | 6.7 | 98.19 | 95.05 | 93.17 |
| Q6_2 | 23,073,522 | 6.92 | 21,689,227 | 6.51 | 98.42 | 95.52 | 94 |
| Q6_3 | 26,711,412 | 8.01 | 24,796,802 | 7.44 | 98.28 | 95.22 | 92.83 |
| Q6+Z7_1 | 23,894,951 | 7.17 | 22,211,718 | 6.66 | 98.3 | 95.27 | 92.96 |
| Q6+Z7_2 | 23,244,747 | 6.97 | 21,524,396 | 6.46 | 98.27 | 95.18 | 92.6 |
| Q6+Z7_3 | 22,896,338 | 6.87 | 21,157,847 | 6.35 | 98.24 | 95.13 | 92.41 |

**Table S4. Expression level of related genes of marine *A. alternata* Z4 and terrestrial *A. alternata* Q6 under coculture conditions with** ***N. sphaerica* Z7** **compared to monoculture.**

| **gene_id** | **Z4+Z7vsZ4** | | **Q6+Z7vsQ6** | | **description** | **abbcrviation** |
| --- | --- | --- | --- | --- | --- | --- |
|  | **log2FC** | **pval** | **log2FC** | **pval** |  |  |
| **biosynthetic gene clusters** | | | | | | |
| Cluster-1287.882 | 4.758445322 | 0.012771 | - | - | Nonribosomal peptide synthetase |  |
| Cluster-1287.9656 | 2.893084796 | 0.028557 | -1.440572591 | 0.028241 | Nonribosomal peptide synthetase |  |
| Cluster-1213.0 | 2.827532804 | 0.040457 | - | - | Nonribosomal peptide synthetase |  |
| Cluster-298.0 | 2.560714954 | 0.015723 | - | - | Nonribosomal peptide synthetase |  |
| Cluster-1287.2291 | 1.950833943 | 0.00000061 | -0.825669819 | 0.0074928 | Nonribosomal peptide synthetase |  |
| Cluster-1287.8984 | 1.402098444 | 0.047002 | - | - | Nonribosomal peptide synthetase |  |
| Cluster-871.0 | 1.395484702 | 0.0066588 | - | - | Nonribosomal peptide synthetase |  |
| Cluster-1287.2966 | 1.235954872 | 0.00000397 | -1.004024257 | 2.53E-10 | Nonribosomal peptide synthetase |  |
| Cluster-1287.9187 | 1.146841388 | 0.0075088 | - | - | Nonribosomal peptide synthetase |  |
| Cluster-1287.9062 | 1.087462841 | 0.008818 | - | - | Nonribosomal peptide synthetase |  |
| Cluster-1082.2 | 0.930737338 | 0.006293 | -3.485426827 | 0.046258 | Nonribosomal peptide synthetase |  |
| Cluster-1287.7438 | 0.806900554 | 0.024456 | -0.309991475 | 0.0078776 | Nonribosomal peptide synthetase |  |
| Cluster-951.1 | 0.700439718 | 0.016171 | - | - | Nonribosomal peptide synthetase |  |
| Cluster-1287.1634 | 0.626238776 | 0.0093957 | - | - | Nonribosomal peptide synthetase |  |
| Cluster-1287.5607 | 0.573185333 | 0.012468 | -0.694997046 | 9.75E-08 | Nonribosomal peptide synthetase |  |
| Cluster-1287.7630 | 0.328421857 | 0.0016048 | -1.111682789 | 1.39E-12 | Nonribosomal peptide synthetase |  |
| Cluster-1287.1353 | 0.297680549 | 0.024136 | - | - | Nonribosomal peptide synthetase |  |
| Cluster-1287.2115 | 0.11783649 | 0.022465 | -1.584962501 | 0.0097081 | Nonribosomal peptide synthetase |  |
| Cluster-1157.0 | 3.257387843 | 0.01947 | - | - | Polyketide synthase |  |
| Cluster-840.0 | 2.705256734 | 0.0060317 | -3.142957954 | 0.031306 | Polyketide synthase |  |
| Cluster-1287.846 | 2.510961919 | 0.0060309 | - | - | Polyketide synthase |  |
| Cluster-1287.3033 | 1.101086125 | 0.0059171 | -0.764371752 | 0.0077641 | Polyketide synthase |  |
| Cluster-1157.1 | 1.062464087 | 0.0080923 | - | - | Polyketide synthase |  |
| Cluster-1287.7067 | 1.059109807 | 0 | -0.071516874 | 0.0092261 | Polyketide synthase |  |
| Cluster-1287.2592 | 0.588275235 | 0.009128 | -0.531523242 | 0.017 | Polyketide synthase | *pksI* |
| Cluster-1287.7213 | 0.475760493 | 0.011161 | -0.612810486 | 0.0017389 | Polyketide synthase |  |
| TCA cycle | | | | | | |
| Cluster-1287.5959 | 1.299061722 | 1.5456E-06 | 0.142933408 | 0.000011947 | pyruvate dehydrogenase E2 component (dihydrolipoyllysine-residue acetyltransferase) | *pdhC* |
| Cluster-1287.5441 | 1.249381145 | 6.6119E-06 | 0.059554176 | 0.0010968 | isocitrate dehydrogenase (NAD+) | *IDH3* |
| Cluster-1287.5134 | 1.15787966 | 18628E-06 | -0.448238204 | 0.00029828 | pyruvate carboxylase | *PC* |
| Cluster-1287.7559 | 1.093966308 | 4.9682E-10 | 0.046725317 | 0.016359 | ATP citrate (pro-S)-lyase | *ACLY* |
| Cluster-1287.4503 | 1.026884351 | 0.00083707 | -0.11589479 | 0.073494 | 2-oxoglutarate dehydrogenase E1 component | *sucA* |
| Cluster-1287.4994 | 1.008020693 | 1.9601E-06 | 0.217486899 | 2.1425E-06 | pyruvate dehydrogenase E1 component subunit beta | *pdhB* |
| Cluster-1287.6980 | 0.870066365 | 0.000018339 | 0.068091266 | 0.0019818 | pyruvate dehydrogenase E1 component subunit alpha | *pdhA* |
| Cluster-1287.6039 | 0.800656174 | 0.014413 | -0.333195833 | 4.8005E-06 | phosphoenolpyruvate carboxykinase (ATP) | *pckA* |
| Cluster-1287.6376 | 0.718924648 | 0.023457 | 0.17916292 | 2.2088E-08 | aconitate hydratase | *ACO* |
| Cluster-1287.5365 | 0.676535712 | 0.042849 | 0.044345597 | 0.015317 | succinate dehydrogenase (ubiquinone) membrane anchor subunit | *SDHD* |
| Cluster-1287.5598 | 0.626342323 | 0.027073 | -1.030463986 | 1.1123E-45 | aconitate hydratase | *ACO* |
| Cluster-1287.6051 | 0.563733626 | 0.0092567 | 0.088356725 | 0.0013101 | succinyl-CoA synthetase alpha subunit | *LSC1* |
| Cluster-1287.7442 | 0.559859891 | 0.0035075 | -0.088693702 | 0.0052765 | succinyl-CoA synthetase beta subunit | *LSC2* |
| Cluster-1287.6541 | 0.529474724 | 0.043738 | -0.596631273 | 3.5668E-27 | malate dehydrogenase | *MDH2* |
| Cluster-1287.4752 | 0.489700969 | 0.034939 | 0.067040677 | 0.0067957 | succinate dehydrogenase (ubiquinone) iron-sulfur subunit | *SDHB* |
| Cluster-1287.6890 | 0.462226264 | 0.0088063 | -0.053472131 | 0.013393 | succinate dehydrogenase (ubiquinone) flavoprotein subunit | *SDHA* |
| Glycolysis / Gluconeogenesis | | | | | | |
| Cluster-1287.6808 | 3.294620749 | 0.0069979 | 0.16756388 | 1.8756E-06 | fructose-bisphosphate aldolase, class II | *fbaA* |
| Cluster-1287.6709 | 1.165950143 | 0.024777 | 0.036796819 | 0.00085867 | phosphoglycerate kinase | *pgk* |
| Cluster-1287.5745 | 1.144995761 | 6.7114E-07 | 0.001687165 | 0.025762 | 2,3-bisphosphoglycerate-independent phosphoglycerate mutase | *gpmI* |
| Cluster-1287.7500 | 1.026581693 | 0.000080386 | -0.686988211 | 8.7868E-21 | aldehyde dehydrogenase (NAD+) | *ALDH* |
| Cluster-1287.4994 | 1.008020693 | 1.9601E-06 | 0.217486899 | 2.1425E-06 | pyruvate dehydrogenase E1 component subunit beta | *pdhB* |
| Cluster-1287.7090 | 0.871415794 | 0.0059285 | -0.665081067 | 0.013928 | aldehyde dehydrogenase (NAD+) | *ALDH* |
| Cluster-1287.6980 | 0.870066365 | 0.000018339 | 0.068091266 | 0.0019818 | pyruvate dehydrogenase E1 component subunit alpha | *pdhA* |
| Cluster-1287.1376 | 0.804449191 | 0.0060192 | -1.336525471 | 0.015001 | aldehyde dehydrogenase (NAD+) | *ALDH* |
| Cluster-1287.6039 | 0.800656174 | 0.014413 | -0.333195833 | 4.8005E-06 | phosphoenolpyruvate carboxykinase (ATP) | *pckA* |
| Cluster-1287.4455 | 0.657463243 | 0.064655 | -0.250390197 | 0.020191 | hexokinase | *HK* |
| Cluster-1287.4585 | 0.656521634 | 0.011023 | -0.325361849 | 0.001277 | hexokinase | *HK* |
| Cluster-1287.6499 | 0.609045141 | 0.0067155 | -0.663267002 | 2.4825E-29 | phosphoglucomutase | *pgm* |
| Cluster-1287.8443 | 0.56828376 | 0.027263 | 0.172180975 | 0.016215 | aldehyde dehydrogenase (NAD+) | *ALDH* |
| Cluster-1287.5660 | 0.564895754 | 0.0096989 | -0.216805921 | 0.0097144 | fructose-1,6-bisphosphatase I | *fbp* |
| Cluster-1287.7541 | 0.531324537 | 0.0084628 | -0.455882552 | 0.011939 | hexokinase | *HK* |
| Cluster-1287.6684 | 0.514685586 | 0.018251 | 0.204207253 | 2.8068E-07 | fructose-bisphosphate aldolase, class II | *fbaA* |
| Cluster-1287.8629 | 0.191772779 | 0.064959 | -1.695675724 | 1.0189E-08 | fructose-bisphosphate aldolase, class II | *fbaA* |
| Cluster-1287.6850 | 0.08404073 | 0.00015738 | 0.163722213 | 1.3715E-06 | glyceraldehyde 3-phosphate dehydrogenase (phosphorylating) | *GAPDH* |
| Cluster-1287.6826 | 0.027974299 | 0.00006113 | -0.355189285 | 4.8651E-08 | enolase | *eno* |
| Oxidative phosphorylation | | | | | | |
| Cluster-1287.7828 | 1.501567463 | 2.5659E-12 | 0.159681663 | 3.7342E-06 | NADH:quinone reductase (non-electrogenic) | *ndh* |
| Cluster-1287.1612 | 1.399607459 | 0.0076877 | -3.139551352 | 0.000032357 | choline transport protein | *CTR* |
| Cluster-1287.7871 | 1.178837916 | 9.34E-08 | -0.257087513 | 0.018611 | multisite-specific tRNA:(cytosine-C5)-methyltransferase | *NCL1* |
| Cluster-1287.2258 | 1.169925001 | 0.0053805 | -1.823122238 | 0.034937 | ubiquinol-cytochrome c reductase cytochrome b subunit | *CYTB* |
| Cluster-1287.4269 | 1.068609899 | 0.0074982 | -0.655056225 | 0.026092 | cytochrome c oxidase assembly protein subunit 23 | *COX23* |
| Cluster-1287.4820 | 1.058270605 | 0.023064 | -0.180645277 | 0.0061734 | NADH dehydrogenase | *NDUFAF6* |
| Cluster-1287.7590 | 1.005759269 | 0.000047195 | -0.291819247 | 0.013116 | phosphatidylinositol phospholipase C, delta | *PLCD* |
| Cluster-1287.6816 | 1.004590513 | 1.5591E-13 | 0.950357304 | 1.0632E-76 | yeast amino acid transporter | *YAT* |
| Cluster-1287.6303 | 0.905883673 | 0.000056132 | -0.437348604 | 9.7555E-06 | myosin V | *MYO5* |
| Cluster-1287.8096 | 0.877914361 | 0.01434 | -0.288055406 | 0.012958 | NADH dehydrogenase | *NDUFAF1* |
| Cluster-1287.6651 | 0.814372453 | 0.0090111 | -0.764063085 | 0.0039623 | NADH dehydrogenase (ubiquinone) 1 alpha subcomplex subunit 13 | *NDUFA13* |
| Cluster-1287.3532 | 0.785082371 | 0.021501 | -0.466644376 | 0.013077 | cytochrome c oxidase assembly protein subunit 11 | *COX11* |
| Cluster-1287.6138 | 0.712670358 | 0.010982 | 0.177833653 | 1.7913E-06 | NADH dehydrogenase (ubiquinone) Fe-S protein 1 | *NDUFS1* |
| Cluster-1287.7323 | 0.685838583 | 0.044576 | -0.478077201 | 0.015242 | NADH dehydrogenase (ubiquinone) Fe-S protein 6 | *NDUFS6* |
| Cluster-1287.4312 | 0.680323597 | 0.0081896 | -0.29969706 | 0.0086807 | NADH dehydrogenase | *NDUFAF2* |
| Cluster-1287.5365 | 0.676535712 | 0.042849 | 0.044345597 | 0.015317 | succinate dehydrogenase (ubiquinone) membrane anchor subunit | *SDHD* |
| Cluster-1287.6720 | 0.620486725 | 0.040046 | -0.347234489 | 0.0025652 | 4-coumarate--CoA ligase | *4CL* |
| Cluster-1287.7465 | 0.512317199 | 0.0085074 | 0.079909074 | 0.008554 | ribonuclease P/MRP protein subunit POP1 | *POP1* |
| Cluster-1287.4752 | 0.489700969 | 0.034939 | 0.067040677 | 0.0067957 | succinate dehydrogenase (ubiquinone) iron-sulfur subunit | *SDHB* |
| Cluster-1287.5992 | 0.485328987 | 0.049956 | 0.111102247 | 0.00050576 | NADH dehydrogenase (ubiquinone) Fe-S protein 2 | *NDUFS2* |
| Cluster-1287.1489 | 0.479609501 | 0.098445 | -1.614709844 | 0.087831 | cytochrome c oxidase subunit 1 | *COX1* |
| Cluster-1287.5300 | 0.469453925 | 0.058537 | -0.237129833 | 0.050952 | L-lactate dehydrogenase (cytochrome) | *lldD* |
| Cluster-1287.6507 | 0.467604836 | 0.49629 | 0.361908792 | 5.393E-12 | ubiquinol-cytochrome c reductase core subunit 2 | *QCR2* |
| Cluster-1287.3080 | 0.46610066 | 0.83337 | -0.74271053 | 0.029929 | mannan polymerase II complex MNN10 subunit | *MNN10* |
| Cluster-1287.6890 | 0.462226264 | 0.88063 | -0.053472131 | 0.013393 | succinate dehydrogenase (ubiquinone) flavoprotein subunit | *SDHA* |
| Cluster-1287.4248 | 0.449673841 | 0.76067 | -0.077342089 | 0.77572 | release factor glutamine methyltransferase | *N6AMT1* |
| Cluster-1287.7043 | 0.427947334 | 0.012876 | -0.160199148 | 0.066823 | H+-transporting ATPase | *PMA1* |
| Cluster-1287.7057 | 0.425755052 | 0.55791 | 0.180345425 | 0.00079942 | NADH dehydrogenase (ubiquinone) 1 alpha subcomplex subunit 8 | *NDUFA8* |
| Cluster-1287.5623 | 0.400141287 | 0.90108 | -0.147337053 | 0.73505 | forkhead protein FKH | *FKH* |
| Cluster-1287.7134 | 0.393949307 | 0.69738 | 0.207266908 | 1.081E-09 | ubiquinol-cytochrome c reductase cytochrome c1 subunit | *CYC1* |
| Cluster-1287.6481 | 0.37805297 | 0.43293 | -0.152656449 | 0.16426 | ubiquinol-cytochrome c reductase subunit 7 | *QCR7* |
| Cluster-1287.8179 | 0.364712961 | 0.48617 | -0.243823802 | 0.53511 | transcription initiation factor TFIID subunit 9B | *TAF9B* |
| Cluster-1287.6248 | 0.361602721 | 0.074257 | 0.085468427 | 0.001885 | cytochrome c oxidase subunit 7c | *COX7C* |
| Cluster-1287.3616 | 0.357731439 | 0.58114 | 0.01110022 | 0.11092 | alpha-1,3-glucosyltransferase | *ALG6* |
| Cluster-1287.6173 | 0.352883392 | 0.45916 | 0.282925787 | 2.2961E-09 | heme a synthase | *COX15* |
| Cluster-1287.3999 | 0.348965664 | 0.72115 | -0.336857054 | 0.032042 | NADH dehydrogenase | *NDUFAF5* |
| Cluster-1287.5394 | 0.33390683 | 0.23947 | -0.077543838 | 0.14181 | ubiquinol-cytochrome c reductase subunit 8 | *QCR8* |
| Cluster-1287.6799 | 0.330092402 | 0.9151 | 0.011781015 | 0.11237 | magnesium transporter | *ALR* |
| Cluster-1287.5478 | 0.313353065 | 0.02819 | 0.122045744 | 0.000001695 | cytochrome c oxidase subunit 4 | *COX4* |
| Cluster-1287.6238 | 0.30603179 | 0.92323 | -0.359343396 | 0.50221 | NADH dehydrogenase (ubiquinone) 1 alpha subcomplex subunit 2 | *NDUFA2* |
| Cluster-1287.7426 | 0.286249119 | 0.059053 | 0.010124217 | 0.030019 | gephyrin | *GPHN* |
| Cluster-1287.6179 | 0.264639315 | 0.12701 | -0.130822159 | 0.79376 | 20S proteasome subunit alpha 5 | *PSMA5* |
| Cluster-1287.118 | 0.263034406 | 0.02134 | -2.700439718 | 0.24346 | cytochrome c oxidase subunit 3 | *COX3* |
| Cluster-1287.5030 | 0.231410313 | 0.082027 | -0.274950539 | 0.16577 | H+-transporting ATPase | *PMA1* |
| Cluster-1287.6406 | 0.177626862 | 0.022664 | 0.188339808 | 2.931E-07 | NADH dehydrogenase (ubiquinone) flavoprotein 1 | *NDUFV1* |
| Cluster-1287.5982 | 0.147025233 | 0.019247 | 0.160011929 | 0.000041456 | cytochrome c oxidase subunit 4 | *COX4* |
| Cluster-1287.3301 | 0.001956197 | 0.0026086 | 0.112751293 | 0.010485 | acetylcholinesterase | *ACHE* |
| MAPK signaling pathway | | | | | | |
| Cluster-1287.9077 | 2.40599236 | 0.044465 | -1.797507136 | 0.031417 | serine/threonine-protein kinase HSL1, negative regulator of Swe1 kinase | *HSL1* |
| Cluster-1287.4056 | 1.482047269 | 0.000032241 | -0.282671399 | 0.030227 | pheromone receptor transcription factor | *MCM1* |
| Cluster-1287.5287 | 1.334419039 | 0.00067997 | -0.596584868 | 0.024094 | tyrosine-protein phosphatase MSG5 | *MSG5* |
| Cluster-1287.6101 | 1.306192268 | 0.0074874 | 0.033486433 | 0.022759 | general transcriptional corepressor CYC8 | *CYC8* |
| Cluster-1287.3479 | 1.2891668 | 0.000050355 | -0.003252978 | 0.010582 | cyclin-dependent kinase | *CDC28* |
| Cluster-1287.4178 | 1.22209922 | 1.2883E-08 | 0.170226096 | 0.00051186 | SHO1 osmosensor | *SHO1* |
| Cluster-1287.4227 | 1.088471687 | 0.00012693 | 0.259643817 | 1.7883E-07 | mitogen-activated protein kinase kinase kinase | *STE11* |
| Cluster-1287.4516 | 1.088314072 | 2.5412E-06 | -0.255592472 | 0.17598 | serine/threonine-protein kinase CLA4 | *CLA4* |
| Cluster-1287.6106 | 1.08136304 | 0.0046191 | 0.18735123 | 4.5236E-09 | transcription factor STE12 | *STE12* |
| Cluster-1287.633 | 1.060047384 | 0.010134 | - | - | catalase | *katE* |
| Cluster-1287.4861 | 1.048398143 | 0.0047467 | -0.01651793 | 0.00041287 | 1-phosphatidylinositol-4-phosphate 5-kinase | *PIP5K* |
| Cluster-1287.5930 | 1.039018578 | 0.0098082 | -0.32111782 | 0.0077945 | 1,3-beta-glucan synthase | *E2.4.1.34* |
| Cluster-1287.8682 | 1.0369134 | 0.000040629 | -0.026152288 | 0.023622 | glycerol-3-phosphate dehydrogenase (NAD+) | *GPD1* |
| Cluster-1287.3572 | 1.001440534 | 0.000020207 | 0.058588324 | 0.054153 | type II protein arginine methyltransferase | *HSL7* |
| Cluster-1287.6772 | 0.937093248 | 0.039944 | -0.373927997 | 0.066018 | phosphatidylinositol 4-kinase A | *PI4KA* |
| Cluster-1287.5759 | 0.812708157 | 0.13104 | 0.107902878 | 0.0030521 | transcription factor RLM1 | *RLM1* |
| Cluster-1287.5892 | 0.790805201 | 0.026734 | 0.058893689 | 0.00013219 | ATF/CREB family transcription factor | *SKO1* |
| Cluster-1287.4073 | 0.781450475 | 0.063059 | 0.047500424 | 0.011677 | p21-activated kinase 1 | *PAK1* |
| Cluster-1287.4156 | 0.764875881 | 0.7349 | - | - | RNA polymerase II-associated factor 1 | *PAF1* |
| Cluster-1287.7174 | 0.764018514 | 0.0058923 | 0.230005605 | 2.9627E-07 | mitosis inhibitor protein kinase SWE1 | *SWE1* |
| Cluster-1287.5317 | 0.691750444 | 0.0351 | -0.177380806 | 0.0056479 | cell division control protein 42 | *CDC42* |
| Cluster-1287.4948 | 0.645581139 | 0.034038 | 0.078002512 | 0.073329 | regulatory protein SWI6 | *SWI6* |
| Cluster-1287.6165 | 0.639443996 | 0.007846 | 0.036276792 | 0.0013818 | cell division control protein 24 | *CDC24* |
| Cluster-1287.5468 | 0.539458307 | 0.0098664 | 0.003021352 | 0.028143 | p38 MAP kinase | *hog1* |
| Cluster-1287.8015 | 0.491022726 | 0.0063178 | 0.065015565 | 0.0086885 | mitogen-activated protein kinase kinase | *STE7* |
| Cluster-1287.4469 | 0.486343552 | 0.015437 | 0.118881762 | 0.0011398 | protein OPY2 | *OPY2* |
| Cluster-1287.7017 | 0.482532084 | 0.044159 | 0.213983602 | 7.433E-08 | GTPase-activating protein SST2 | *SST2* |
| Cluster-1287.4005 | 0.476272751 | 0.0061411 | -0.169961441 | 0.044939 | mitogen-activated protein kinase kinase | *MKK1_2* |
| Cluster-1287.5579 | 0.432521535 | 0.02251 | 0.438762131 | 2.8551E-13 | bud emergence protein 1 | *BEM1* |
| Cluster-1287.7540 | 0.425290198 | 0.045694 | -0.334722534 | 0.079529 | RHO1 GDP-GTP exchange protein 1/2 | *ROM1_2* |
| Cluster-1287.6485 | 0.394012928 | 0.0083284 | 0.082030445 | 0.00017604 | mitogen-activated protein kinase kinase | *PBS2* |
| Cluster-1287.7054 | 0.374428438 | 0.031059 | 0.060703303 | 0.0015535 | mitogen-activated protein kinase kinase kinase | *SSK2* |
| Cluster-1287.6437 | 0.349876323 | 0.036493 | -0.188502213 | 0.065865 | cell wall integrity and stress response component | *WSC* |
| Cluster-1287.6475 | 0.290807683 | 0.025427 | 0.268817431 | 4.1465E-12 | 14-3-3 protein epsilon | *YWHAE* |
| Cluster-1287.7194 | 0.240832389 | 0.005454 | 0.047066217 | 0.043911 | protein phosphatase PTC1 | *PTC1* |
| Cluster-1287.5397 | 0.232493011 | 0.009892 | 0.019000962 | 0.0026267 | osomolarity two-component system, sensor histidine kinase SLN1 | *SLN1* |
| Cluster-1287.3825 | 0.163653453 | 0.020945 | -0.057274805 | 0.071163 | pheromone a factor receptor | *STE3* |
| Cluster-1287.2684 | 0.15935576 | 0.036759 | -0.698619283 | 0.2932 | pheromone a factor receptor | *STE3* |
| Cluster-1287.7079 | 0.110988795 | 0.0016225 | -0.074998752 | 0.12525 | tyrosine-protein phosphatase 2/3 | *PTP2_3* |
| Cluster-1287.4473 | 0.017585366 | 0.00016967 | 0.013709924 | 0.10154 | osomolarity two-component system, response regulator SSK1 | *SSK1* |
| Amino acid metabolism | | | | | | |
| Cluster-1287.4028 | -0.832566866 | 9.5568E-10 | -3.397400064 | 1.3853E-234 | alcohol dehydrogenase (NADP+) | *adh* |
| Cluster-1287.3898 | 1.31410859 | 0.028557 | -2.487938046 | 4.8751E-18 | primary-amine oxidase | *tynA* |
| Cluster-1287.4069 | 0.905801358 | 0.0088041 | -1.708382636 | 5.0415E-16 | phenylacetate 2-hydroxylase | *PHAA* |
| Cluster-955.0 | - | - | -1.523561956 | 0.006274 | monoamine oxidase | *MAO* |
| Cluster-1287.1376 | 0.804449191 | 0.060192 | -1.336525471 | 0.015001 | aldehyde dehydrogenase (NAD+) | *ALDH* |
| Cluster-1287.73 | - | 0.69001 | -1.164386818 | 0.0063349 | ATP-binding cassette, subfamily G (WHITE), member 2, PDR | *PDR* |
| Cluster-1287.4304 | 0.046355636 | 0.0039787 | -1.102264932 | 4.7936E-21 | primary-amine oxidase | *tynA* |
| Cluster-1287.7993 | 0.332403539 | 0.022842 | -1.073581132 | 0.0081241 | alcohol dehydrogenase, propanol-preferring | *adhP* |
| Cluster-1287.2340 | 0.231456504 | 0.0066225 | -0.842208754 | 0.0097773 | alcohol dehydrogenase, propanol-preferring | *adhP* |
| Cluster-1287.1649 | 0.344510503 | 0.25087 | -0.768435933 | 0.023408 | alcohol dehydrogenase, propanol-preferring | *adhP* |
| Cluster-1287.6927 | 0.906425885 | 0.067057 | -0.707819249 | 0.026291 | alcohol dehydrogenase, propanol-preferring | *adhP* |
| Cluster-1287.7500 | 1.046101734 | 0.000080386 | -0.686988211 | 8.7868E-21 | aldehyde dehydrogenase (NAD+) | *ALDH* |
| Cluster-1287.7090 | 1.006126103 | 0.59285 | -0.665081067 | 0.013928 | aldehyde dehydrogenase (NAD+) | *ALDH* |
| Cluster-1287.3918 | 0.420055574 | 0.24432 | -0.654214947 | 0.000017809 | primary-amine oxidase | *tynA* |
| Cluster-1287.2843 | 0.235155373 | 0.10117 | -0.634350528 | 0.05843 | primary-amine oxidase | *tynA* |
| Cluster-1287.7176 | 0.13689227 | 0.02338 | -0.102138191 | 0.0086026 | alcohol dehydrogenase (NADP+) | *adh* |
| Carbohydrate metabolism | | | | | | |
| Cluster-1287.1640 | 3.025535092 | 0.0064867 | -1.584962501 | 0.0070699 | pectinesterase | *E3.1.1.11* |
| Cluster-1287.1348 | 2.179323699 | 0.0070087 | -2.141355849 | 0.033543 | galacturan 1,4-alpha-galacturonidase | *E3.2.1.67* |
| Cluster-1287.8416 | 1.043195868 | 0.001051 | -1.0710878 | 2.1306E-16 | L-glyceraldehyde reductase | *GAAD* |
| Cluster-1287.2630 | 0.740240726 | 0.008714 | -3.291188746 | 0 | L-glyceraldehyde reductase | *GAAD* |
| Cluster-1287.4893 | 0.612495684 | 0.14916 | -0.129837438 | 0.97614 | polygalacturonase | *E3.2.1.15* |
| Cluster-1287.2200 | 0.499571009 | 0.019621 | - | 0.96488 | galacturan 1,4-alpha-galacturonidase | *E3.2.1.67* |
| Cluster-1287.2336 | 0.307258406 | 0.055672 | -0.672678646 | 0.016815 | pectinesterase | *E3.1.1.11* |
| Cluster-1287.4010 | 0.101173679 | 0.0019697 | -0.454918368 | 0.0080512 | polygalacturonase | *E3.2.1.15* |
| Laccase | | | | | | |
| Cluster-1287.4424 | 1.248820547 | 2.3834E-09 | -0.450661409 | 0.0072558 | Laccase-1 |  |
| Cluster-1287.1476 | 1.246639968 | 0.0094403 | -1.016301812 | 0.0053031 | Laccase-3 |  |
| Cluster-1287.2053 | 1.11345805 | 0.0072827 | -0.317266718 | 0.0084633 | Laccase-3 |  |
| Cluster-1287.1684 | 1.028569152 | 0.00661 | 0.569195185 | 0.000015504 | Laccase-2 |  |
| Cluster-1287.8694 | 0.790676181 | 0.0074445 | -0.260025656 | 0.0045989 | Iron transport multicopper oxidase | *FET3_5* |
| Cluster-1287.3269 | 0.566650622 | 0.0052536 | -0.189079264 | 0.0074836 | Laccase-like |  |
| Heat shock protein | | | | | | |
| Cluster-1287.5282 | 1.758597685 | 3.5878E-23 | -0.342304231 | 0.000045117 | heat shock protein 90 | *HSP90A, htpG* |
| Cluster-1287.7611 | 1.698787336 | 5.5389E-20 | -0.04954069 | 0.17193 | Heat shock protein | *DNAJA2* |
| Cluster-1287.7605 | 1.491723503 | 1.0359E-22 | -0.235512436 | 0.070082 | Heat shock protein hsp88 | *HSPA4* |
| Cluster-1287.5883 | 1.448903502 | 1.8261E-14 | -0.535651277 | 1.5771E-09 | heat shock 70 kda protein | *HSPA1_6_8* |
| Cluster-1287.7241 | 1.316382115 | 9.389E-13 | 0.065520394 | 0.0020116 | heat shock protein 70 | *HSPA9* |
| Cluster-1287.7355 | 1.281747811 | 1.6183E-16 | 0.364150715 | 2.5245E-12 | Heat shock protein 60 | *HSPD1* |
| Cluster-1287.4590 | 1.273410159 | 3.4276E-06 | 0.071285363 | 0.021566 | Heat shock protein |  |
| Cluster-1287.5221 | 1.246780145 | 1.3599E-13 | 0.518716545 | 5.0235E-27 | heat shock protein-like protein | *HSPA1_6_8* |
| Cluster-1287.4860 | 1.060182673 | 1.0493E-07 | -0.086608567 | 0.042988 | hsp70-like protein |  |
| Cluster-1287.8230 | 1.035046947 | 0.000071228 | -0.012443063 | 0.030147 | Heat shock protein | *dnaJ* |
| Cluster-1287.8260 | 1.018581426 | 0.00010029 | -0.463246592 | 0.12804 | Heat shock protein |  |
| Cluster-1287.8032 | 1.009984089 | 0.047849 | 0.005271087 | 0.001808 | Heat shock protein |  |
| Cluster-1287.5584 | 0.829130684 | 0.0042039 | -0.173067675 | 0.0067503 | Heat shock protein 78 | *clpB* |
| Cluster-1287.7508 | 0.759420445 | 0.010156 | -0.258683523 | 0.042727 | Heat shock protein | *HYOU1* |
| Cluster-1287.5999 | 0.722831628 | 0.0099056 | -0.101351489 | 0.024579 | heat shock protein 70 | *HSPA5* |
| Cluster-1287.6310 | 0.648834684 | 0.013733 | 0.03829652 | 0.015859 | Heat shock protein |  |
| Cluster-1287.1219 | 0.632268215 | 0.021318 | -1.928446739 | 0.01784 | Heat shock protein |  |
| Cluster-1287.2451 | 0.534113359 | 0.0066835 | -0.197708158 | 0.0073087 | Heat shock protein |  |
| Cluster-1287.4013 | 0.531925554 | 0.025909 | -0.047320494 | 0.0052847 | Heat shock protein | *SCJ1* |
| Cluster-1287.3913 | 0.491313267 | 0.0067433 | -0.10648457 | 0.0058751 | Heat shock protein 83-1 |  |
| Cluster-1287.4393 | 0.364980338 | 0.033172 | -1.801167999 | 2.3006E-61 | 30 kDa heat shock protein | *HSP20* |
| Cluster-1287.4989 | 0.310878615 | 0.013726 | 0.047104904 | 0.015406 | Heat shock factor protein 2 | *HSFF* |
| Cluster-1287.4573 | 0.231099117 | 0.011652 | -0.213657611 | 0.0083792 | Heat shock protein |  |
| Cluster-1287.5503 | 0.21146562 | 0.015311 | 0.02116818 | 0.02444 | Heat shock protein |  |
| Cluster-1287.5861 | 0.17746604 | 0.031859 | -0.493427166 | 2.9353E-06 | dnaj heat shock family protein | *DNAJB4* |
| Basal transcription factors | | | | | | |
| Cluster-1287.4540 | 1.296845345 | 0.000034708 | -0.324456921 | 0.068854 | transcription initiation factor TFIIE subunit alpha | *TFIIE1* |
| Cluster-1287.5656 | 1.185228326 | 4.1011E-10 | -0.069078383 | 0.065685 | cyclin-dependent kinase 7 | *CDK7* |
| Cluster-1287.4584 | 1.177121378 | 0.0011116 | -0.243211248 | 0.017844 | transcription initiation factor TFIID subunit 12 | *TAF12* |
| Cluster-1287.2729 | 1.142759249 | 0.10461 | -0.564551041 | 0.0099485 | DNA excision repair protein ERCC-2 | *ERCC2* |
| Cluster-1287.5524 | 1.049592682 | 0.13609 | -0.040878221 | 0.01937 | transcription initiation factor TFIID subunit 6 | *TAF6* |
| Cluster-1287.4680 | 1.02685122 | 0.015885 | -0.540110833 | 0.079317 | transcription initiation factor TFIID subunit 13 | *TAF13* |
| Cluster-1287.6016 | 1.007249739 | 0.0032923 | -0.220035076 | 0.03097 | transcription initiation factor TFIID subunit 1, fungi type | *TAF1* |
| Cluster-1287.6108 | 1.001884644 | 0.00030764 | -0.266658829 | 0.14552 | bromodomain-containing factor 1 | *BDF1* |
| Cluster-1287.7303 | 0.918288657 | 0.036307 | -0.423162687 | 0.13006 | transcription initiation factor TFIIH subunit 1 | *TFIIH1* |
| Cluster-1287.8006 | 0.865270401 | 0.011208 | -0.122241253 | 0.72664 | transcription initiation factor TFIID subunit 8 | *TAF8* |
| Cluster-1287.3848 | 0.8105432 | 0.000029049 | -0.445547407 | 0.00020397 | transcription initiation factor TFIID subunit 5 | *TAF5* |
| Cluster-1287.5526 | 0.716051073 | 0.090542 | - | - | histone H2B | *H2B* |
| Cluster-1287.6148 | 0.708489721 | 0.0086912 | -0.017505109 | 0.10669 | transcription initiation factor TFIIF subunit alpha | *TFIIF1* |
| Cluster-1287.4051 | 0.630417522 | 0.0085853 | 0.341079792 | 1.7994E-09 | transcription initiation factor TFIID subunit 15 | *TAF15* |
| Cluster-1287.5615 | 0.605344001 | 0.019482 | -0.590752181 | 1.1066E-08 | DNA excision repair protein ERCC-3 | *ERCC3* |
| Cluster-1287.8086 | 0.519764004 | 0.0082803 | 0.078333747 | 0.019977 | transcription initiation factor TFIID/TFIIF subunit | *TAF14* |
| Cluster-1287.7245 | 0.42014841 | 0.051597 | -0.221424494 | 0.07523 | transcription initiation factor TFIID subunit 7 | *TAF7* |
| Cluster-1287.8179 | 0.364712961 | 0.48617 | -0.243823802 | 0.053511 | transcription initiation factor TFIID subunit 9B | *TAF9B* |
| Cluster-1287.7798 | 0.303999539 | 0.0069791 | 0.280296761 | 3.7859E-09 | transcription initiation factor TFIID TATA-box-binding protein | *TBP* |
| Cluster-1287.5135 | 0.265885138 | 0.027164 | -0.471949944 | 4.7871E-07 | transcription initiation factor TFIID subunit 3 | *TAF3* |
| Cluster-1287.7558 | 0.16416671 | 0.0084738 | -0.090386236 | 0.01315 | transcription initiation factor TFIID subunit 2 | *TAF2* |
| Cluster-1287.6764 | 0.14100191 | 0.053849 | -0.078484841 | 0.13435 | transcription initiation factor TFIIH subunit 2 | *TFIIH2* |
| Cluster-1287.6537 | 0.301328326 | 0.018481 | 0.461807629 | 5.3212E-11 | Secondary metabolism regulator LAE1 | *LaeA* |
| Cluster-1287.7189 | 0.741005793 | 0.0058545 | 0.553591687 | 4.5751E-29 | Developmental and secondary metabolism regulator VEL1 | *VeA* |
| Cluster-1287.2538 | 1.145979306 | 0.0072985 | 0.006562688 | 0.0091981 | DNA-binding transcription factor activity,Fungal Zn(2)-Cys(6) binuclear cluster domain | *aohR* |
| Oxidative stress response | | | | | | |
| Cluster-1287.214 | 1.091147888 | 0.007854 | -0.97924144 | 0.0050083 | catalase | *katE* |
| Cluster-1287.633 | 1.060047384 | 0.010134 | - | - | catalase | *katE* |
| Cluster-1287.7650 | 1.029146346 | 0.00032075 | -0.101399449 | 0.036496 | sodium/hydrogen antiporter | *SOD2* |
| Cluster-1287.6823 | 0.829603352 | 0.0069276 | 0.326768251 | 2.1102E-14 | superoxide dismutase, Cu-Zn family | *SOD1* |
| Cluster-1287.5969 | 0.623617912 | 0.0063437 | -0.340950037 | 0.0023921 | NADPH oxidase 1 | *NOX1* |
| Cluster-1287.6369 | 0.565424253 | 0.035826 | -0.483923979 | 0.000062703 | osomolarity two-component system, response regulator SKN7 | *SKN7* |
| Cluster-1287.2435 | 0.311461096 | 0.025467 | -0.748461233 | 0.035144 | sodium/hydrogen antiporter | *SOD2* |
| Cluster-1287.5329 | 0.188445089 | 0.0092263 | -0.200139614 | 0.0054274 | catalase | *katE* |
| Cluster-1287.8401 | 0.129767875 | 0.015459 | 0.038006323 | 0.0075635 | sodium/hydrogen antiporter | *SOD2* |
| Cluster-1287.3904 | 0.040930264 | 0.034137 | -0.219631282 | 0.006499 | catalase | *katE* |
| Cluster-1287.4998 | 0.654022341 | 0.0055998 | -0.024662054 | 0.047979 | Ferrous iron transport protein B | *APC1* |
| Cluster-1287.7549 | 0.728060041 | 0.00015591 | -0.170833786 | 0.1484 | iron ion binding | *infB* |
| glutathione metabolism | | | | | | |
| Cluster-1287.8536 | 0.759213428 | 0.01193 | -0.176537825 | 0.021748 | gamma-glutamyltranspeptidase / glutathione hydrolase | *ggt* |
| Cluster-1287.6219 | 0.754801548 | 6.7674E-08 | -0.355130671 | 8.5137E-06 | glucose-6-phosphate 1-dehydrogenase | *G6PD* |
| Cluster-1287.7652 | 0.636218159 | 0.014116 | -0.446240999 | 0.0056814 | importin-11 | *IPO11* |
| Cluster-1287.4931 | 0.608762035 | 0.16253 | -0.189590461 | 0.0078139 | glutathione synthase | *GSS* |
| Cluster-1287.7919 | 0.597726192 | 0.0071159 | -0.112272874 | 0.0070257 | glutamate--cysteine ligase regulatory subunit | *GCLM* |
| Cluster-1287.6607 | 0.506202768 | 0.0056181 | -0.510647934 | 5.7908E-07 | gamma-glutamyltranspeptidase / glutathione hydrolase | *ggt* |
| Cluster-1287.6801 | 0.400331 | 0.03356 | 0.006091614 | 0.0040721 | 5-oxoprolinase (ATP-hydrolysing) | *OPLAH* |
| Cluster-1287.6861 | 0.236124304 | 0.087394 | -0.15918728 | 0.043186 | glutathione reductase (NADPH) | *GSR* |
| Cluster-1287.5067 | 0.183525086 | 0.028693 | -0.346571833 | 0.0020217 | 5-oxoprolinase (ATP-hydrolysing) | *OPLAH* |
| Cluster-1287.7842 | 0.115775572 | 0.010406 | 0.058089507 | 0.041827 | glutamate--cysteine ligase catalytic subunit | *GCLC* |
| DNA repair | | | | | | |
| Cluster-1287.8356 | 1.582494245 | 3.1629E-06 | -0.18420624 | 0.036178 | DNA polymerase phi | *POL5* |
| Cluster-1287.8384 | 1.523061207 | 2.8206E-10 | -0.462464117 | 0.0026485 | mitochondrial inner membrane protease subunit 1 | *IMP1* |
| Cluster-1287.5590 | 1.303000813 | 0.016381 | -0.692857242 | 0.0040154 | DNA polymerase delta subunit 3 | *POLD3* |
| Cluster-1287.3272 | 1.209568381 | 0.05851 | -0.207904658 | 0.0035302 | DNA polymerase epsilon subunit 2 | *POLE2* |
| Cluster-1287.3299 | 1.004661377 | 0.013616 | -0.377264157 | 0.054035 | DNA polymerase alpha subunit B | *POLA2* |
| Cluster-1287.8271 | 0.993544978 | 0.02684 | 0.277533976 | 0.00065937 | DNA polymerase gamma 1 | *POLG* |
| Cluster-1287.7499 | 0.945712926 | 0.0014375 | -0.11911414 | 0.0068717 | DNA polymerase iota | *POLI* |
| Cluster-1287.8506 | 0.889639553 | 0.004758 | -0.078355638 | 0.014179 | DNA polymerase eta | *POLH* |
| Cluster-1287.3716 | 0.762669054 | 0.00079029 | -0.241029054 | 0.06905 | UV excision repair protein RAD23 | *RAD23* |
| Cluster-1287.8240 | 0.737923877 | 0.029759 | -0.517009472 | 0.000011742 | DNA polymerase alpha subunit A | *POLA1* |
| Cluster-1287.7437 | 0.588784212 | 0.0086407 | -0.419619627 | 0.0001765 | DNA polymerase delta subunit 1 | *POLD1* |
| Cluster-1287.3664 | 0.561311233 | 0.0068095 | -0.465974465 | 0.039996 | DNA polymerase epsilon subunit 1 | *POLE* |
| Cluster-1287.6318 | 0.560801971 | 0.020748 | -0.306047295 | 0.000023757 | serine/threonine-protein kinase ATR | *ATR* |
| Cluster-1287.4418 | 0.542053909 | 0.0091409 | -0.750771394 | 0.071749 | serine-protein kinase ATM | *ATM* |
| Cluster-1287.7046 | 0.378261467 | 0.0071259 | -0.34886581 | 0.069785 | DNA repair and recombination protein RAD54B | *RAD54B* |
| Cluster-1287.7964 | 0.292180751 | 0.023898 | -0.094704424 | 0.01985 | DNA repair protein REV1 | *REV1* |
| Cluster-1287.1791 | 0.282933963 | 0.49316 | 0.062464087 | 0.03279 | DNA polymerase IV | *POL4* |
| Cluster-1287.4836 | 0.273997187 | 0.021519 | -0.321630908 | 0.013372 | flap endonuclease-1 | *RAD2* |
| Cluster-1287.7667 | 0.238340492 | 0.076956 | 0.003796568 | 0.020071 | DNA excision repair protein ERCC-5 | *RAD2* |
| Cluster-1287.8580 | 0.221172383 | 0.019909 | 0.344911413 | 0.012044 | DNA polymerase zeta | *POLZ* |
| Cluster-1287.5433 | 0.036224713 | 0.00025106 | 0.124519022 | 0.00007061 | DNA repair protein RAD7 | *RAD7* |
